# Metabolic alterations in immune cells associate with progression to type 1 diabetes

**DOI:** 10.1007/s00125-020-05107-6

**Published:** 2020-02-11

**Authors:** Partho Sen, Alex M. Dickens, María Asunción López-Bascón, Tuomas Lindeman, Esko Kemppainen, Santosh Lamichhane, Tuukka Rönkkö, Jorma Ilonen, Jorma Toppari, Riitta Veijola, Heikki Hyöty, Tuulia Hyötyläinen, Mikael Knip, Matej Orešič

**Affiliations:** 1 Turku Bioscience Centre, University of Turku and Åbo Akademi University, FI-20520 Turku, Finland; 2 Department of Analytical Chemistry, University of Córdoba, Córdoba, Spain; 3 Department of Chemistry, Örebro University, Örebro, Sweden; 4 Immunogenetics Laboratory, Institute of Biomedicine, University of Turku, Turku, Finland; 5 Clinical Microbiology, Turku University Hospital, Turku, Finland; 6 Department of Pediatrics and Adolescent Medicine, Turku University Hospital, Turku, Finland; 7 Institute of Biomedicine, Centre for Integrative Physiology and Pharmacology, University of Turku, Turku, Finland; 8 Department of Pediatrics, PEDEGO Research Unit, Medical Research Centre, University of Oulu, Oulu, Finland; 9 Department of Children and Adolescents, Oulu University Hospital, Oulu, Finland; 10 Department of Women’s and Children’s Health, Karolinska Institutet, Stockholm, Sweden; 11 Faculty of Medicine and Life Sciences, University of Tampere, Tampere, Finland; 12 Fimlab Laboratories, Pirkanmaa Hospital District, Tampere, Finland; 13 Children’s Hospital, University of Helsinki and Helsinki University Hospital, 00290 Helsinki, Finland; 14 Research Program for Clinical and Molecular Metabolism, Faculty of Medicine, University of Helsinki, Helsinki, Finland; 15 Tampere Centre for Child Health Research, Tampere University Hospital, Tampere, Finland; 16 School of Medical Sciences, Örebro University, Örebro, Sweden

**Keywords:** Birth cohort, Ceramides, Genome-scale metabolic modelling, Lipidomics, Metabolomics, Peripheral blood mononuclear cells, Sphingolipid metabolism, Type 1 diabetes

## Abstract

**Aims/hypothesis:**

Previous metabolomics studies suggest that type 1 diabetes is preceded by specific metabolic disturbances. The aim of this study was to investigate whether distinct metabolic patterns occur in peripheral blood mononuclear cells (PBMCs) of children who later develop pancreatic beta cell autoimmunity or overt type 1 diabetes.

**Methods:**

In a longitudinal cohort setting, PBMC metabolomic analysis was applied in children who (1) progressed to type 1 diabetes (PT1D, *n* = 34), (2) seroconverted to ≥1 islet autoantibody without progressing to type 1 diabetes (P1Ab, *n* = 27) or (3) remained autoantibody negative during follow-up (CTRL, *n* = 10).

**Results:**

During the first year of life, levels of most lipids and polar metabolites were lower in the PT1D and P1Ab groups compared with the CTRL group. Pathway over-representation analysis suggested alanine, aspartate, glutamate, glycerophospholipid and sphingolipid metabolism were over-represented in PT1D. Genome-scale metabolic models of PBMCs during type 1 diabetes progression were developed by using publicly available transcriptomics data and constrained with metabolomics data from our study. Metabolic modelling confirmed altered ceramide pathways, known to play an important role in immune regulation, as specifically associated with type 1 diabetes progression.

**Conclusions/interpretation:**

Our data suggest that systemic dysregulation of lipid metabolism, as observed in plasma, may impact the metabolism and function of immune cells during progression to overt type 1 diabetes.

**Data availability:**

The GEMs for PBMCs have been submitted to BioModels (www.ebi.ac.uk/biomodels/), under accession number MODEL1905270001. The metabolomics datasets and the clinical metadata generated in this study were submitted to MetaboLights (https://www.ebi.ac.uk/metabolights/), under accession number MTBLS1015.

**Electronic supplementary material:**

The online version of this article (10.1007/s00125-020-05107-6) contains peer-reviewed but unedited supplementary material, which is available to authorised users.

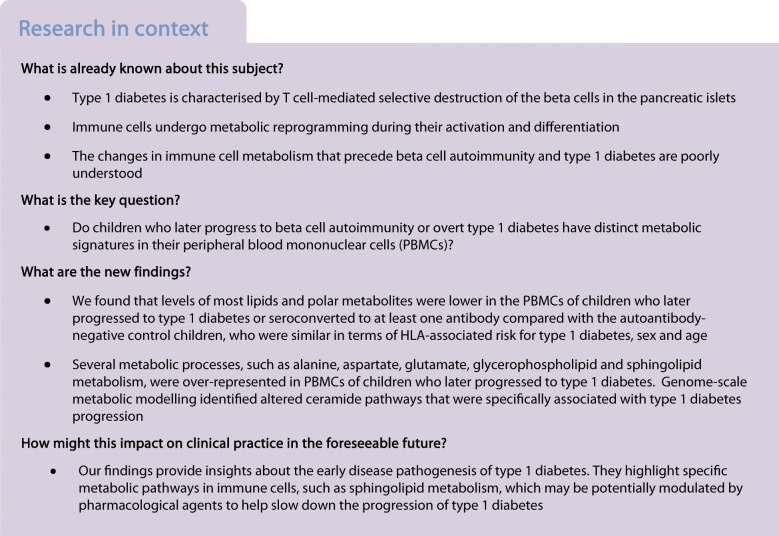



## Introduction

The incidence of type 1 diabetes in most Western countries has been increasing over the past few decades, particularly among children below 5 years of age [1]. About 70% of children with type 1 diabetes carry increased risk-associated genotypes in HLA loci, whereas only 3–7% of the population with the same risk alleles develop type 1 diabetes [2].

The appearance of autoantibodies against insulin (IAA), a 65 kDa isoform of GAD (GADA), insulinoma-associated antigen-2 (IA-2A), and/or zinc transporter 8 (ZnT8A) in the plasma is an early sign of emerging islet autoimmunity and clinical type 1 diabetes [3]. It is known that children with multiple islet autoantibodies in particular have an increased risk of type 1 diabetes [4]. In addition to genetic predisposition, other exogenous environmental factors affect risk, such as intestinal dysbiosis, reduced gut microbial diversity [5], level of hygiene [6] and infant-feeding regimen [7, 8] are implicated in the initiation of beta cell autoimmunity. Our recent data also suggest that prenatal exposure to environmental chemicals modulates lipid metabolism in newborn infants and increases their subsequent risk of type 1 diabetes [9]. However, the early pathogenesis of type 1 diabetes is still poorly understood and of the molecular signatures and related pathways predictive of progression to overt type 1 diabetes have yet to be identified.

Alterations in immune cell metabolism may affect the host immune system [10]. In fact, external perturbation of key metabolic processes, such as glycolysis and amino acid metabolism, have already been shown to impair T cell activation, differentiation and cytokine production [11]. Human peripheral blood mononuclear cells (PBMCs), including T cells (~70%), B cells (~15%), monocytes (~5%), dendritic cells (~1%) and natural killer (NK) cells (~10%) obtained from healthy donors and progressors to type 1 diabetes are already being investigated in order to better understand this phenomenon [12]. Such efforts seek to elucidate how immune cell metabolic processes are altered in seroconversion and progression to overt type 1 diabetes; currently a largely unknown area.

Metabolomics is the study of small (<1500 Da) molecules and their functions in cells, tissues and body fluids [13]. The metabolome, which can be seen partly as a phenotypic readout of the genome, is sensitive to changes in immune system status, diet and the gut microbiota [14]. Through metabolomic analyses, we have previously shown that decreased levels of plasma sphingomyelins (SMs) and phosphatidylcholines (PCs) are associated with progression to type 1 diabetes [15–17].

In this study, we applied metabolomics to determine levels of molecular lipids and polar metabolites in PBMCs isolated from prospective samples collected in the Type 1 Diabetes Prediction and Prevention (DIPP) study, with the aim of elucidating the events preceding the onset of islet autoimmunity and overt type 1 diabetes. We sought to address whether distinct metabolic patterns can be discerned during infancy among three study groups of children: (1) those who developed clinical type 1 diabetes, (2) those who seroconverted to at least one islet autoantibody but were not diagnosed with type 1 diabetes during follow-up and (3) a control group, i.e. children who remained autoantibody negative during follow-up.

## Methods

### Study design and protocol

In this study, the samples were obtained from the Finnish DIPP study [18]. The DIPP study has screened more than 230,000 newborn infants for HLA-conferred susceptibility to type 1 diabetes in three university hospitals: those at Turku, Tampere and Oulu in Finland [19]. The children involved in the current study were chosen from the subset of DIPP children which were from the city of Tampere, Finland. The study protocol was approved by the ethics and research committee of University of Tampere and Tampere University Hospital. The study was conducted according to the guidelines of the Declaration of Helsinki. Written informed consent was provided by the parents at the beginning of the study for the children to participate in the study. Here, longitudinal samples for each child were collected between 1998 and 2012. For each child, longitudinal samples for PBMC metabolomic analysis were obtained at 12, 24 and 36 months of age.

This study comprises samples (*n* = 137 for lipidomics and *n* = 134 for polar metabolites) from 71 children, divided into three groups:: (1) 27 children who seroconverted to at least one islet autoantibody but were not diagnosed with type 1 diabetes during the follow-up period (P1Ab), (2) 34 children who seroconverted to more than one islet autoantibody and subsequently developed type 1 diabetes (PT1D), and (3) ten control children (CTRL), i.e. children who remained islet autoantibody negative during follow-up. The three study groups were similar in terms of HLA-associated risk for type 1 diabetes, sex and age. Selected characteristics of the participants involved in this study are listed in (Table 1**)**.

**Table 1:** Demographic and clinical characteristics of the study population

Characteristic	CTRL	P1Ab	PT1D
Number of participants	10	27	34
Sex (male, female) (*n*)	(6, 4)	(16, 11)	(10, 24)
Age at time of diagnosis (months, median ± SD)	–	–	53.0 ± 30.48
Age of first seroconversion (months, median ± SD)	–	24.0 ± 20.12	14.0 ± 6.13
HLA risk^a^ (*n*)			
High risk	3	3	6
Moderate risk	1	14	17
Low or neutral risk	6	10	11

### HLA genotyping

Screening for HLA-conferred susceptibility to type 1 diabetes was performed using cord blood samples. The HLA genotyping was performed using a time-resolved, fluorometry-based assay for four alleles using lanthanide chelate-labelled, sequence-specific oligonucleotide probes detecting DQB1*02, DQB1*03:01, DQB1*03:02, and DQB1*06:02/3 alleles [20]. The carriers of genotypes DQB1*02/DQB1*03:02 or DQB1*03:02/x genotypes (here x = DQB1*03:01, DQB1*06:02, or DQB1*06:03 alleles) were categorised as being eligible and recruited for the DIPP follow-up programme in Tampere until 3 years of age.

A more extensive HLA genotyping was performed for the children participating this study. This genotyping defined all common European HLA-DR-DQ haplotypes at low resolution and at higher resolution haplotypes where this was relevant for estimation of the risk for type 1 diabetes conferred, e.g. HLA-DR4 subtypes in DR4-DQ8 haplotypes. In a series of 2991 family trios from the Finnish Pediatric Diabetes Register, the genotype risks were defined and genotypes were combined into six groups from (strongly protective) to 5 (high risk) which did not overlap for 95% CIs of their OR values for type 1 diabetes [21].

### Detection of islet autoantibodies

The children with HLA-conferred genetic susceptibility were prospectively observed for levels of type 1 diabetes-associated autoantibodies (ICA, IAA, IA-2A and GADA). These autoantibodies were assayed from plasma samples taken at each follow-up visit as previously described [22]. Levels of islet cell autoantibodies were determined using an approved immunofluorescence assay with a detection limit of 2.5 Juvenile Diabetes Foundation Units (JDFU) [23]. GADA and IAA levels were quantified using specific radiobinding assays, the threshold of positivity being 5.36 and 3.48 relative units (RU), respectively [24, 25]. Similarly, IA-2A levels were measured with a radiobinding assay with a threshold of 0.43 RU [26].

### Analysis of molecular lipids and polar metabolites

In this study, non-fasting blood samples were collected, plasma was prepared within 3 h of sample collection and stored at −80°C until analysed (see electronic supplementary material [ESM] Methods for further details). The samples were randomised and extracted using a modified version of the previously published Folch procedure [27, 28]. Molecular lipids were determined using ultra-high-performance liquid chromatography-quadrupole time-of-flight mass spectrometry (UHPLC-Q-TOF-MS). Identification of lipids was carried out by combining MS (and retention time), MS/MS information and a search of the LIPID MAPS spectral database (http://www.lipidmaps.org).

For determination of polar metabolites, the samples were derivatised using a two-step procedure. Initially the samples were methoximated by incubating the samples with methoxyamine hydrochloride (25 μL, 20 mg/ml in pyridine, Sigma-Aldrich, Chemie, Taufkirchen, Germany) at 45°C for 1 h. *N*-Methyl-*N*-(trimethylsilyl)trifluoroacetamide (MSTFA, 25 μl, Sigma-Aldrich) was then added and the samples were incubated for a further 1 h. A retention index standard containing straight chain, even alkanes (*n* 10–40, 10 μl, Sigma-Aldrich) was added. The derivatised samples were analysed using gas chromatography (Agilent 7890B, Agilent Technologies, Santa Clara, CA, USA) coupled to a single quad mass spectrometer (5977B). Further details of the analysis of molecular lipids and polar metabolites in the PBMCs, along with the data pre-processing can be found in the ESM Methods.

### Statistical methods

The lipidomics and polar metabolites datasets were divided into three study groups: CTRL, P1Ab, and PT1D (Table 1, ESM Fig. 1). The age of the participant was calculated as the time difference between the date the sample was withdrawn and the date of birth of the child. If more than two samples from the same child matched a time interval, the closest was selected. Each group was divided into three age groups of 12, 24 and 36 months (ESM Fig. 1). The data were log_2_-transformed. Homogeneity of the samples was assessed by principal component analysis (PCA) [29] and no outliers were detected (95% CI). The log_2_-normalised intensities of the total identified lipids and polar metabolites in the participants are shown in ESM Figs 2 and 3, respectively. The differences in PBMC lipidomes and polar metabolites between the study groups (P1Ab vs CTRL, PT1D vs CTRL, PT1D vs P1Ab), at 12, 24 and 36 months of age, were explored independently by using multivariate analysis (sparse partial least squares discriminant analysis [sPLS-DA]) [30] and univariate analysis (unpaired two-sample *t* test) (see ESM Statistical Methods). The R statistical programming language [31] was used for data analysis and visualisation. Further details of data analysis, including pathway over-representation analysis (POA), packages and software are discussed in the ESM.

### Meta-analysis of transcriptomics datasets and genome-scale metabolic modelling

In order to understand the regulation of metabolic pathways in PBMCs after seroconversion and type 1 diabetes progression, genome-scale metabolic models (GEMs) [12, 32–34] of PBMCs were developed. Gene expression or transcriptomics datasets were used to contextualise these models for the P1Ab, P1TD and CTRL groups. Gene expression data of PBMCs was obtained from two related cohorts: (1) BABYDIET [35–37], a prospective birth cohort of children being studied for the progression to islet autoimmunity and type 1 diabetes and (2) Diabetes-Genes, Autoimmunity and Prevention (D-GAP) study, a prospective study that recruited children newly diagnosed with type 1 diabetes [37]. The longitudinal study settings of these cohorts are similar to the DIPP study design [3]. The datasets from these studies were downloaded from ArrayExpress (www.ebi.ac.uk; accession number E-MTAB-1724). Expression data for 15 non-progressors (P1Ab), 51 cases of type 1 diabetes (PT1D), and their controls (CTRL) were selected for genome-scale metabolic modelling (GSMM) [12, 32–34]. In addition, differential expression of genes (DEG) for P1Ab vs CTRL, PT1D vs CTRL and PT1D vs P1Ab groups was performed. The *p* values and log_2_ fold changes were calculated.

A GEM for PBMCs was developed by applying the INIT algorithm [38] on Human Metabolic Reconstruction (HMR 2.0) [39] as a template model. GEMs were contextualised/constrained for different conditions using expression datasets. The gene/transcript expression data obtained from PBMCs of PT1D, P1Ab and CTRL were employed to score each reaction of HMR 2.0. Contextualisation and analysis of GEMs for PBMCs are further described in the ESM.

## Results

### Global lipidome of immune cells in progression to islet autoimmunity and type 1 diabetes

PBMCs were isolated from children who (1) progressed to clinical type 1 diabetes during follow-up (PT1D, *n* = 34), (2) seroconverted to at least one islet autoantibody but were not diagnosed with type 1 diabetes during follow-up (P1Ab, *n* = 27) or (3) remained autoantibody negative during follow-up (CTRL, *n* = 10) (Table 1). The lipidomics dataset comprised 153 lipid species. Sources of variation in the PBMC lipidome dataset were identified using linear regression modelling, where the concentrations of lipids were regressed with various clinical variables such as age, sex, disease conditions and their interactions. This analysis showed that the age of an individual indeed had a confounding effect (>10% of explained variation, EV) on the lipidome (ESM Fig. 4). The effect of sex was, however, minimal (<1% EV) (ESM Fig. 4). The interactions between or among the factors had no significant effect on the lipidome.

**Fig. 1:**
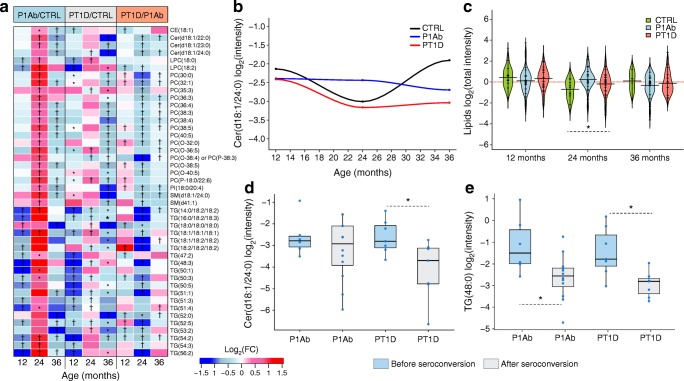
Lipid profiles in PBMCs during follow-up. (**a**) Log_2_ fold changes (FCs) of lipid levels in P1Ab vs CTRL, PT1D vs CTRL and PT1D vs P1Ab at 12, 24 and 36 months of age. ^†^ denotes changes in lipid levels between the groups (sPLS-DA: AUC ~0.65, RC >±0.05, VIP scores >1 [40]); and unpaired two-sample *t* test: *p* value <0.05), as evaluated by univariate and multivariate analyses; * denotes changes (*p* < 0.05) in lipid levels between groups, as evaluated by univariate analysis (unpaired two-sample *t* test) only. Red, blue and white colour spectrum signifies up-, downregulation and no change, respectively. CE, cholesterol ester; PI, phosphatidylinositol. (**b**) Log_2_ mean intensities of Cer(d18:1/24:0) across different age groups. Loess regression was used for the interpolation of the data points. The number of participants (‘*n*’) of a particular group, at a particular age included in this analysis is shown in ESM Fig. 1. (**c**) Intensities of total lipids in CTRL, P1Ab and PT1D groups at 12, 24 and 36 months of age. The red dotted line denotes the mean of the population. The black dashes in the bean plots represent individual participants and their corresponding lipid levels, and the extended black lines denote the group mean. Mean of the subgroups at a particular age were compared by ANOVA *p < 0.05. (**d**, **e**) Boxplots showing the levels of Cer(d18:1/24:0) and TG(48:0) in participants (indicated by the blue dots), before and after the seroconversion. Black horizontal lines in the boxplots indicate the median log intensities of Cer(d18:1/24:0) and TG(48:0) in each group, the boxes represent the first to third quartiles. A paired *t* test was performed as a test of significance, *p < 0.05

The results from multi- and univariate analyses suggest that lipid levels in PBMCs from the P1Ab and PT1D groups are different from those in PBMCs from the CTRL group (Fig. 1a**)**. Many classes of lipids, including cholesterol esters (CEs), lysophosphatidylcholines (LPCs), PCs, phosphatidylethanolamines (PEs), phosphatidylinositols (PIs), SMs, ceramides (Cers) and triacylglycerols (TGs) were altered (sPLS-DA: AUC ~0.65, regression coefficient [RC] (>±0.05), variable importance in projection [VIP] scores >1 [40]) and/or (unpaired two-sample *t* test: *p* < 0.05) between these groups (Fig. 1a).

At 12 months of age, i.e. before the median age of seroconversion, the levels of some TG, PC, LPC and Cer species were lower in the PBMCs of the P1Ab and PT1D groups, as compared with those in the PBMCs of the CTRL group (Fig. 1a). At 24 months of age, there was a subtle increase or no change in these same lipid levels in the P1Ab and PT1D groups (Fig. 1a, c, ESM Fig. 5). This effect was most prominently seen in the P1Ab group, where the total lipid level was higher (*p* = 0.048) than in the CTRL group (Fig. 1c). Interestingly, this accumulation was transient, as these lipids had returned to their previous (12 month time point) levels by 36 months (Fig. 1a, c). However, Cer(d18:1/24:0) was persistently decreased in the PT1D group compared with the P1Ab and CTRL groups (Fig. 1b). The log mean intensities of selected lipids across different age groups are shown in **(**ESM Fig. 5). In this analysis, time was not explicitly considered for statistical analysis, as the follow-up samples from all the participants were not available at all time points.

### PBMC lipidome before and after the first appearance of islet autoantibodies

We aimed to identify the molecular lipids that were altered (*p* < 0.05) following seroconversion to islet autoimmunity (vs before seroconversion) in both the P1Ab and PT1D groups (Fig. 1d). Cer(d18:1/24:0) and TGs with low carbon number and double bond count were downregulated in PT1D after seroconversion (ESM Table 1). Total lipids in the P1Ab group (*p* = 6 × 10^−5^) and PT1D group (*p* = 1 × 10^−5^) were decreased after seroconversion (ESM Fig. 6).

### Polar metabolites of immune cells in progression to islet autoimmunity and type 1 diabetes

**Fig. 2:**
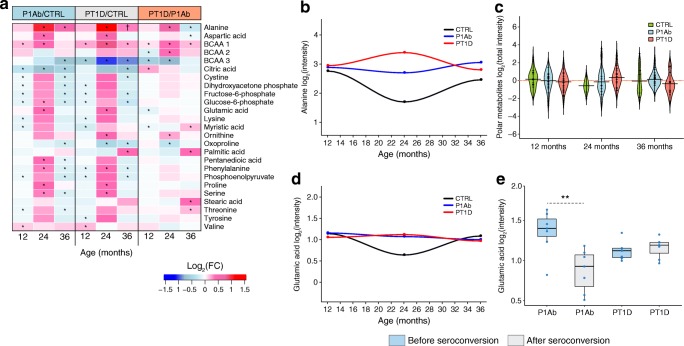
Polar metabolite profiles in PBMCs during follow-up. (**a**) Log_2_ fold changes (FCs) in the levels of the polar metabolites in P1Ab vs CTRL, PT1D vs CTRL and PT1D vs P1Ab groups at 12, 24 and 36 months of follow-up; ^†^ denotes changes in the metabolite levels between the groups (sPLS-DA: AUC ~0.65, RC >±0.05, VIP scores >1 [40]), and unpaired two-sample *t* test: *p* < 0.05), as evaluated by univariate and multivariate analyses; * denotes changes (*p* < 0.05) in the metabolite levels between groups, as evaluated by univariate analysis (unpaired two-sample *t* test) only. Red, blue and white colours signify up-, downregulation and no change respectively. (**b**) Log_2_ mean intensities of alanine in the CTRL, P1Ab and PT1D groups across different age groups during follow-up. Loess regression was used for the interpolation of the data points. The number of participants (‘*n*’) of a particular group, at a specific age included in this analysis is shown in ESM Fig. 1. (**c**) Log_2_ intensities of total polar metabolites as measured in CTRL, P1Ab and PT1D groups at 12, 24 and 36 months of age. The red dotted line denotes the mean of the population. The black dashes in the bean plots represent individual participants and their corresponding levels of total metabolites. The extended black line denotes the group mean. Mean of the subgroups at a particular age were compared by ANOVA. (**d**) Log_2_ mean intensities of glutamic acid in the CTRL, P1Ab and PT1D groups across different age groups during follow-up. Loess regression was used for the interpolation of the data points. The number of participants (‘*n*’) of a particular group, at a specific age included in this analysis is shown in ESM Fig. 1. (**e**) Levels of glutamic acid in each participant (indicated by the blue dots), before and after seroconversion; **p < 0.01). Black horizontal lines in the boxplot indicate the median log intensity of glutamic acid in a group, the boxes represent the first to third quartiles. A paired *t* test was performed as a test of significance

We analysed polar metabolites from PBMCs from the same samples as in the lipidomic analyses (ESM Table 2). Using sPLS-DA and univariate analyses, as for the lipidomic analyses, 25 polar metabolites were altered (sPLS-DA: AUC ~0.60, RC (>±0.05), VIP scores >1 [40]) and/or (unpaired two-sample *t* test, *p* value <0.05) between the study groups (P1Ab vs CTRL, PT1D vs CTRL, PT1D vs P1Ab), at 12, 24 and 36 months of age (Fig. 2a**)**. These metabolites can be divided into major chemical classes, including carboxylic acids, amino acids, sugar derivatives, hydroxy acids, phenolic compounds, fatty acids and phosphate derivatives. The log mean intensities of alanine and glutamic acid in the PT1D, P1Ab and CTRL children across different age groups are shown in Fig. 2b and d. Likewise, time was not explicitly considered for statistical analysis, as the follow-up samples from all the participants were not available at all the time points.

At 12 months of age, the majority of the polar metabolites were downregulated in the P1Ab and PT1D groups compared with the CTRL group. At 24 months, there was an increase in the levels of several amino acids including alanine, phenylalanine, proline, serine, threonine, cystine, lysine, glutamic and aspartic acid in the P1Ab and PT1D groups (Fig. 2a, c).

At 36 months of age, i.e. after seroconversion in most children in the PT1D and P1Ab groups, an increase in several saturated fatty acid levels, including stearic, myristic and palmitic acids, was observed in the PT1D group vs the P1Ab group (Fig. 2a). Comparing metabolite levels (ESM Fig. 7) before and after seroconversion, glutamic acid was found to be decreased (*p* = 0.008) after seroconversion in the P1Ab group (Fig. 2e).

### Metabolic associations between immune cells and circulating metabolome

Next, we set out to examine how the metabolite profiles of PBMCs associated with their corresponding plasma profiles. We performed correlation analysis between the metabolites that were altered in the PBMCs across the three study groups (Figs 1, 2), with their corresponding plasma levels, which have previously been reported [16, 41]. These two previous studies were on an expanded group of individuals, a subsample of which was included in the present study.

**Fig. 3:**
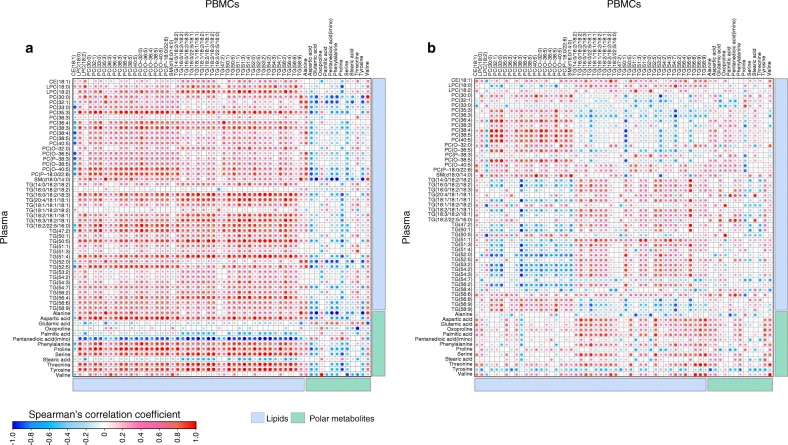
Spearman correlation between plasma (previously reported) and cellular metabolite levels in the CTRL (**a**) and PT1D (**b**) groups at 12 months of age. Red, blue and white colours suggest positive, inverse and no correlation, respectively. CE, cholesterol esters

In the CTRL group, at 12 months of age, the levels of PCs, LPCs, SMs and TGs in PBMCs were positively correlated (Spearman**’**s correlation coefficient, ρ > 0.70, *p* < 0.05) with their corresponding plasma levels (Fig. 3a**)**. Conversely, the polar metabolites were mostly inversely correlated, except alanine, glutamic acid and valine. At this same age, we also found a distinct pattern of PC and TG levels in the PT1D group. Levels of cellular PCs were positively correlated to their corresponding plasma levels, as in the CTRL group, while the same PCs were negatively correlated with specific plasma TG levels (those with a low carbon number and double bond count) (Fig. 3b). Likewise, the levels of plasma PCs were inversely correlated with cellular TG levels. In the P1Ab group, the association between cellular and plasma metabolites exhibited a different pattern: cellular levels of PCs were inversely correlated with their corresponding PC and TG levels in the plasma (ESM Fig. 8).

However, in contrast, at the age of 36 months, the lipid profiles of PBMCs were predominantly positively correlated with their corresponding plasma levels in both the PT1D and P1Ab groups (ESM Figs 8 and 9). These associations were markedly different in the CTRL group, where predominantly inverse correlations between cellular and plasma lipids were observed, except in the case of the majority of the TGs (ESM Fig. 9).

### Over-representation of metabolic pathways in progression to type 1 diabetes

**Fig. 4:**
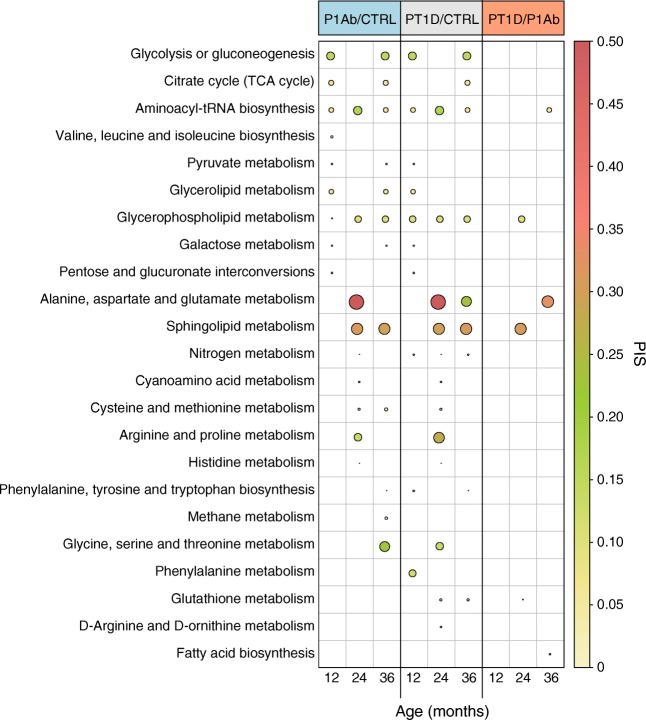
Overrepresentation analysis of metabolic pathways in PBMCs in CTRL, P1Ab and PT1D groups. The plot shows the PIS (false discovery rate [FDR] <0.05) of each metabolic subsystem/pathway during follow-up. Red, green and yellow colours denote high, intermediate and low impacts, respectively. TCA: tricarboxylic acid

The lipids and polar metabolites of PBMCs which differed between the three study groups were mapped against reference human metabolic pathways. The over-represented metabolic subsystems/processes (e.g. glycerolipid metabolism, pyruvate metabolism) were selected based on a false discovery rate (FDR) of <0.05. The pathway impact score (PIS) was estimated for each subsystem (Fig. 4).

Various core metabolic processes were over-represented in the immune cells in the PT1D and/or P1Ab groups (vs CTRL) at 12 months of age, i.e. preceding islet seroconversion and overt type 1 diabetes. These include central carbon metabolism (CCM; e.g. glycolysis, citrate cycle), sugar metabolism, amino acid biosynthesis (valine, leucine and isoleucine; phenylalanine, tyrosine and tryptophan) and glycerophospholipid (GPL) metabolism (Fig. 4**)**. Other metabolic subsystems such as alanine, aspartate and glutamate metabolism (AAG) (PIS ~0.4), sphingolipid metabolism (SMM) (PIS ~0.3), GPL metabolism (PIS ~0.15), arginine and proline metabolism (PIS ~0.25), and aminoacyl-tRNA biosynthesis (PIS ~0.2) were over-represented in the P1Ab and PT1D groups (vs CTRL) at 24 months of age. Moreover, SMM, GPL, AAG, CCM, glycine, serine and threonine (GST) metabolism were either over-represented in P1Ab and/or PT1D groups (vs CTRL) at 36 months of age. Interestingly, at this age, AAG, aminoacyl-tRNA biosynthesis and fatty acid biosynthesis were exclusively over-represented in the PT1D group (vs P1Ab) (Fig. 4**)**.

### Metabolic modelling of sphingolipid metabolism in islet autoimmunity and type 1 diabetes

Given that (1) in our previous study, we observed persistent downregulation of plasma sphingolipids in children who progressed to type 1 diabetes [16, 42], (2) in the present study, SMM was over-represented in PBMCs isolated from the PT1D, and (3) we recently found that prenatal chemical exposure modulates postnatal SM levels and increases type 1 diabetes risk [9], we examined SMM in PBMCs using genome-scale metabolic modelling (ESM Fig. 10). The objective of metabolic modelling, in this case, was to identify the key regulators within SMM in progression to type 1 diabetes. This extended analysis was performed by integrating publicly available transcriptomics datasets obtained from the two related cohorts and the metabolomics dataset from the present study (see Methods). By using the metabolomics dataset from the present study, we devised a confidence score for each metabolic reaction as being either present or absent in the PBMC metabolic model [38]. The model constraints for exchange/input reactions were derived using metabolomics data from the present study.

**Fig. 5:**
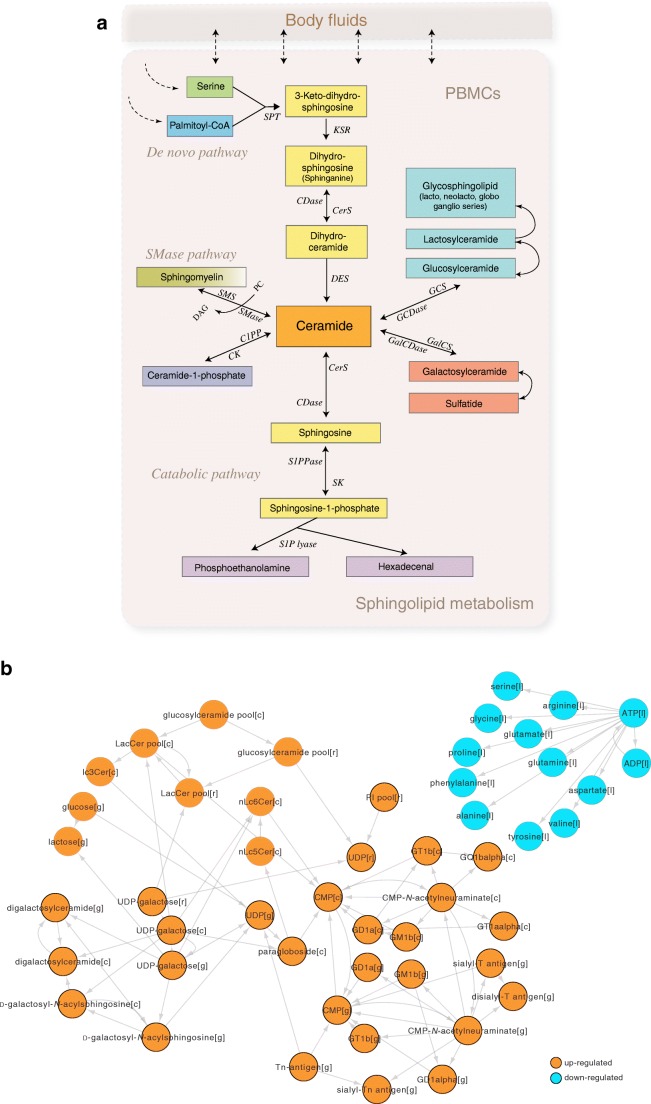
Regulation of sphingomyelin pathways in progression to islet autoimmunity and overt type 1 diabetes. (**a**) Canonical pathways of sphingolipid metabolism in humans. C1PP/C1PPase, ceramide-1-phosphate phosphatase; CDase, ceramidases; CerS, ceramide synthase; CK, ceramide kinase; DAG, diacylglycerol; DES, dihydroceramide desaturase; GalCDase, galactosidase; GalCS, galatosylceramide synthase; GCDase, glucosidase; GCS, glucosylceramide synthase; KSR, 3-keto dihydrosphinganine reductase; PC, phosphatidylcholine; S1P lyase, sphingosine-1-phosphate lyase; S1PPase, sphingosine phosphate phosphatases; SK, sphingosine kinase; SMase, sphingomyelinase; SMS, sphingomyelin synthetase; SPT, serine palmitoyl-CoA transferase. (**b**) RMs predicted for PBMCs that were significantly different (false discovery rate [FDR] <0.05) between PT1D and P1Ab groups. The orange and cyan colours denote up- and downregulation of the RMs, respectively. The cellular compartments ‘[c]’, ‘[g]’, [r]’, ‘[l]’ denote the cytosol, Golgi apparatus, endoplasmic reticulum and lysosome, respectively

Reporter metabolite (RM) [43] analysis showed that glucosyl-, lactosyl- and galactosylceramides were upregulated in the PT1D group compared with P1Ab (Fig. 5b) and CTRL (ESM Fig. 11). These changes were, however, not observed when comparing the P1Ab group with the CTRL group. Instead, LTB4, HETE and EpOME derivatives (all leukotrienes and markers of inflammation) were upregulated (ESM Fig. 12).

**Fig. 6:**
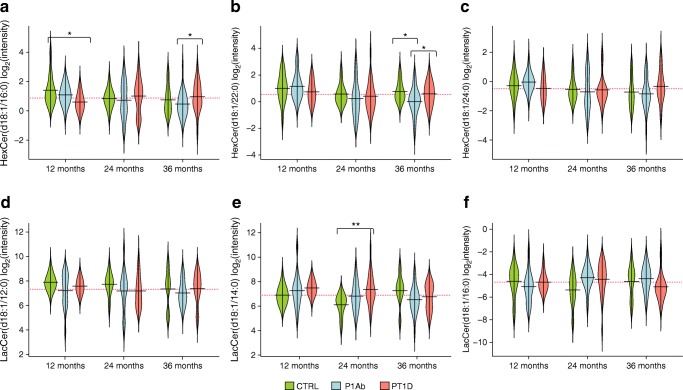
Beanplots showing the levels of hexosylceramides (HexCer) and lactosylceramides (LacCer) in PBMCs. (**a**–**f**) Levels of glycoceramides in the CTRL, P1Ab and PT1D groups at 12, 24 and 36 months. The red dotted line denotes the mean of the population. The black solid lines in the bean plots represent the group mean. *p < 0.05, **p < 0.01 by unpaired two-sample *t* test. The number of participants (‘*n*’) of a particular group, at a particular age included in this analysis is shown in ESM Fig. 1

GSMM of SMM suggests that, in the PT1D group, cellular ceramides are converted to glycoceramides, thus decreasing the free ceramides in the cells*.* In order to confirm these findings, we identified and analysed six glycoceramides from the lipidomics dataset: HexCer(d18:1/16:0), HexCer(d18:1/22:0), HexCer(d18:1/24:0), LacCer(d18:1/12:0), LacCer(d18:1/14:0), LacCer(d18:1/16:0). At 36 months of age, HexCer(d18:1/16:0) and HexCer(d18:1/22:0) were found to be upregulated (*p* < 0.05) in the PT1D group vs the P1Ab group (Fig. 6). There was congruence between the predicted RMs (Fig. 5b) and the glycoceramide levels measured in the PT1D group at 36 months of age. At this age, the ceramides [i.e. Cer(d18:1/24:0) and Cer(d18:1/22:0)] were downregulated in the PT1D vs the P1Ab group (Fig. 1a).

In order to understand the significance of glucosyl-, lactosyl- and digalactosylceramide production, we optimised two key cytoplasmic reactions in SMM, namely, production of glucosylceramides from ceramides and d-glucose (ESM Fig. 13) and formation of digalactosylceramide from *N*-acyl-sphingosine and d-galactose in the three study groups, and thereby recorded the flux changes across the different metabolic subsystems/pathways (ESM Fig. 13). The results suggest that, among several affected metabolic processes, mitochondrial and endoplasmic reticulum transport of substrates might be essential for glycoceramide production. In addition, marked changes in GPL, glycolysis/gluconeogenesis and amino acid metabolism were observed.

## Discussion

We observed metabolic differences in the PBMCs isolated from PT1D, P1Ab and CTRL children. In line with the previous findings in plasma [16, 17, 42, 44], these differences were observed even before the first appearance of islet autoantibodies.

During the first year of life, i.e. before the median age of seroconversion, the majority of lipids and polar metabolites measured in PBMCs were downregulated in the P1Ab and PT1D groups compared with the CTRL group. The occurrence of low levels of TGs of low double bond count and carbon number (i.e. TGs enriched with saturated fatty acids such as palmitate and myristate) suggest an impairment of de novo lipogenesis in PBMCs as being potentially involved in progression towards clinical type 1 diabetes.

At 24 months, i.e. a period coinciding with seroconversion in the majority of children in the PT1D and P1Ab groups, there was an increase in the levels of lipids and amino acids in the PBMCs compared with the control group. There is evidence that amino acids, in particular, branched chain amino acids (BCAAs), contribute to dysregulated lipid metabolism [45]. We observed higher levels of BCAAs and related metabolites, including glutamic acid and alanine, in circulating PBMCs during this period, which is indicative of compromised amino acid catabolism and lipogenesis [46]. In parallel, elevated BCAAs also promote proinflammatory signalling in PBMCs [47]. This observed transient increase in BCAAs in our study suggests that increased BCAAs contribute to immune dysfunction in children who later progress to type 1 diabetes.

There is growing evidence of abnormal SMM in progression to and in early type 1 diabetes [48–50]. Differential analysis of PBMC metabolites in our study showed changes in the levels of ceramides and sphingomyelin species in the PT1D group compared with the CTRL group. Sphingomyelin cleavage and ceramide synthesis is one of the important mechanisms involved in the regulation of immune cell function [51]. Serine and palmitic acid are precursors for de novo sphingomyelin biosynthesis [52]. We found that serine concentrations were decreased in type 1 diabetes progressors after seroconversion. Previous studies by us and others have also reported decreased plasma levels of SMs in children who later progressed to type 1 diabetes and in children with newly diagnosed type 1 diabetes [15, 16, 53]. Our POA provides corroborating evidence that SMM is over-represented in the P1Ab and PT1D groups, suggesting that altered SMM in immune cells, as well as in the circulation, is a hallmark of progression to overt type 1 diabetes.

GSMM predicted that ceramide pathways leading to synthesis of glucosyl-, lactosyl- and galactosylceramides were upregulated in the PT1D group compared with the P1Ab group. This prediction was confirmed by the observed increased levels of these glycosphingolipids in the PT1D group. This strongly implies that ceramides found to be downregulated in the PT1D group of our study are converted to glycoceramides after seroconversion in children who later progress to type 1 diabetes. In agreement with this, we found that the gene expression of glucosylceramide synthase (EC 2.4.1.80), a rate-limiting enzyme in the conversion of ceramides to glucosylceramide and downstream glycosphingolipids, was also increased. Glycoceramides, particularly glucosylceramide, play an important role in the control of immune responses [54]. Previous studies have shown that these glycosphingolipids modulate beta cell immune receptor signalling [55, 56] and aggravate systemic inflammation responses [57, 58]. Elevated glycoceramide levels in PBMCs in autoantibody-positive children who later progress to type 1 diabetes, as observed in our study, therefore point to a specific sphingolipid pathway in immune cells that contributes to type 1 diabetes pathogenesis.

Of note, the current exploratory study provides a catalogue of metabolic signatures that are altered in the human PBMCs obtained from type 1 diabetes progressors and non-progressors, across different age groups. However, the statistical significance of these markers is yet to be validated in a larger cohort, with more samples included per age group.

## Conclusion and future perspectives

Taken together, our results suggest that progression to type 1 diabetes is accompanied by metabolic abnormalities in PBMCs. These changes may be related to impaired de novo lipogenesis, amino acid metabolism, GPL metabolism and SMM. Since specific differences were also observed between progressors and non-progressors to type 1 diabetes after their seroconversion to islet autoimmunity, our findings also highlight specific pathways in immune cells, such as SMM, which appear to play an important role in protection from and progression to type 1 diabetes.

Further mechanistic studies are needed to deconvolute the metabolic response of immune cell subtypes, particularly CD4^+^ and CD8^+^ T cells, B cells and macrophages that represent the full repertoire of the PBMCs [12]. It also remains to be established how the metabolic pathways identified in this study are altered in specific immune cell subtypes, and whether a targeted manipulation of these pathway(s) can suppress an excessive immune response, which, in turn, might retard the onset and/or progression of type 1 diabetes.

## Electronic supplementary material

ESM (PDF 5642 kb)

## Data Availability

The metabolomics datasets and the clinical metadata generated in this study were submitted to MetaboLights [59], under accession number MTBLS1015. Accompanying clinical metadata was linked to the lipidomics dataset using the ISA-creator package from MetaboLights. The GEMs for PBMCs have been submitted to BioModels (www.ebi.ac.uk/biomodels/), under accession number MODEL1905270001.

## Abbreviations

AAG: Alanine, aspartate and glutamate metabolism
BCAA: Branched chain amino acid
CCM: Central carbon metabolism
Cer: Ceramide
CTRL: Children who remained autoantibody negative during follow-up
DIPP: Type 1 Diabetes Prediction and Prevention
GADA: GAD autoantibodies
GEM: Genome-scale metabolic model
GPL: Glycerophospholipid
GSMM: Genome-scale metabolic modelling
IAA: Insulin autoantibodies
IA-2A: Insulinoma-associated antigen-2 autoantibodies
LPC: Lysophosphatidylcholine
P1Ab: Children who seroconverted to ≥1 islet autoantibody without progressing to type 1 diabetes during follow-up
PBMC: Human peripheral blood mononuclear cell
PC: Phosphatidylcholine
PIS: Pathway impact score
POA: Pathway overrepresentation analysis
PT1D: Children who progressed to type 1 diabetes during follow-up
RC: Regression coefficient
RM: Reporter metabolite
SM: Sphingomyelin
SMM: Sphingolipid metabolism
sPLS-DA: Sparse partial least squares discriminant analysis
TG: Triacylglycerol
UPLC-QTOFMS: Ultra-performance liquid chromatography coupled with time-of-flight mass spectrometry
VIP: Variable importance in projection
ZnT8A: Zinc transporter 8 autoantibodies
