# Supplementary material for: Metabolic alterations in immune cells associate with progression to type 1 diabetes
Source: Diabetologia. 2020 Feb 11;63(5):1017–31. doi: 10.1007/s00125-020-05107-6 (PMC7145788; doi:10.1007/s00125-020-05107-6)
Supplement: Supplementary file 1 — (PDF 5642 kb) [file 125_2020_5107_MOESM1_ESM.pdf]

Metabolic Alterations in Immune Cells Associate with Progression to Type 1 Diabetes

Partho Sen *et al.*

Electronic Supplementary Materials (ESM)

Sample preparation

PBMCs were purified using BD Vacutainer® CPT™ Cell Preparation Tube with sodium citrate (Becton, Dickinson and Company, Franklin Lakes, NJ) according to manufacturer's instructions. Collected whole blood was let to cool down for 15-20 minutes in the blood collection tube at room temperature. The blood samples were centrifuged at 3000 g for 20 minutes, after which the layer of mononuclear cells was suspended into the plasma and the suspension was transferred to a new tube. The sample was centrifuged again at 1500 g for 15 minutes. The samples were then divided into cryotubes and snap frozen in liquid nitrogen. The tubes were kept in liquid nitrogen overnight and then stored at -80 °C. For the metabolomic analysis, the samples were thawed on ice and the initial lysing procedures performed in a cold room to prevent changes to the metabolites from the cells or proteins. The cell pellets were resuspended in ice cold saline (0.9% NaCl) by gently pipetting up and down. 25 µL of cells were then aliquoted for metabolomic analysis and stored at -80°C. The total protein content in cells was measured by the Bradford method [1].

Analysis of molecular lipids

The samples were randomized and extracted using a modified version of the previously-published Folch procedure [**2**]. Shortly, 150 µL of 0.9% NaCl was added to the cell pellets, and samples were then vortex mixed and ultrasonicated for 3 minutes. Next, 20 µL of the cell suspension was mixed with 150 µL of the 2.5 µg mL^{-1} internal standards solution in ice-cold CHCl3:MeOH (2:1, v/v). The internal standard solution contained the following compounds: 1,2-diheptadecanoyl-sn-glycero-3-phosphoethanolamine (PE (17:0/17:0)), N-heptadecanoyl-D-erythro-sphingosylphosphorylcholine (SM(d18:1/17:0)), N-heptadecanoyl-D-erythro-sphingosine (Cer(d18:1/17:0)), 1,2-diheptadeca-noyl-sn-glycero-3-phosphocholine (PC(17:0/17:0)), 1-heptadecanoyl-2-hydroxy-sn-glycero-3-phosphocholine (LPC(17:0)) and 1-palmitoyl-d31-2-oleoyl-sn-glycero-3-phosphocholine (PC(16:0/d31/18:1)). These were purchased from Avanti Polar Lipids, Inc. (Alabaster, AL, USA). In addition, triheptadecanoin (TG(17:0/17:0/17:0)) was purchased from (Larodan AB, (Solna, Sweden). The samples were vortex mixed and incubated on ice for 30 min after which they were centrifuged at 7800 g for 5 min. Finally, 60 µL from the lower layer of each sample was collected and mixed with 60 µL of ice-cold CHCl3:MeOH (2:1, *v/v*) in an LC vial. An aliquot of each sample was collected and pooled and used as quality control samples, together with NIST CRM1950 serum sample, an in-house pooled serum sample, pure standard samples and extracted standard samples. In addition, blank samples were analyzed after every 8 samples.

The ultra-high performance liquid chromatography-quadrupole time-of-flight mass spectrometry (UHPLC-Q-TOF-MS) analyses were done in a similar manner to that described earlier, with some modifications [3, 4]. The UHPLC-Q-TOF-MS system was from Agilent Technologies (Santa Clara, CA, USA) combining a 1290 Infinity LC system and 6545 quadrupole time-of-flight mass spectrometer (Q-TOF-MS), interfaced with a dual jet stream electrospray (dual ESI) ion source. MassHunter B.06.01 software (Agilent Technologies, Santa Clara, CA, USA) was used for all data acquisition and MZmine 2 was used for data processing [5].

Chromatographic separation was performed using an Acquity UPLC® BEH C18 column (100 mm × 2.1 mm i.e., 1.7 µm particle size) and protected using a C18 precolumn, both from Waters Corporation (Wexford, Ireland). The injection volume was 1 µL. The mobile phases were water and acetonitrile:2-propanol (1:1, *v*/*v*), both containing 1% 1M ammonium acetate and 0.1% (*v*/*v*) formic acid as ionization agents. The LC pump was programmed at a flow rate of 0.4 mL min^{-1} and the elution gradient was as follows: from min 0–2, the percentage of phase organic phase was modified from 35% to 80%, from min 2-7, the percentage of organic phase was modified from 80–100% and then, the final percentage was held for 7 min. A post-time of 7 min was used to regain the initial conditions for the next analysis. Thus, the total analysis time per sample was 21 min. The column and the eluents were kept at 50 °C and the multisampler was kept at 10°C. The settings of the dual ESI ion source were as follows: capillary voltage 4.5 kV, nozzle voltage 1500 V, N_2 pressure in the nebulizer 21 psi, N_2 flow rate and temperature as sheath gas 11 L min^{-1} and 379 °C, respectively. Accurate mass spectra in the MS scan were acquired in the m/z range 100–1700 in positive ion mode, and in range 100-1700 in the negative mode for the MS/MS runs. In the ESI-mode, capillary voltage 3.6 kV, nozzle voltage 1500 V, N_2 pressure in the nebulizer 21 psi, N_2 flow rate and temperature as sheath gas 11 L min^{-1} and 379 °C, respectively. The injector system was washed with 10% DCM in MeOH and ACN:MeOH:IPA:H2O (1:1:1:1, v/v/v/v) + 0.1% HCOOH as needle wash solutions after each injection for 7.5s each.

Identification of lipids was carried out by combining MS (and retention time), MS/MS information, and a search of the LIPID MAPS spectral database (http://www.lipidmaps.org). MS/MS data were acquired in both negative and positive ion modes in order to maximize identification coverage. The confirmation of a lipid's structure requires the identification of hydrocarbon chains bound to its polar moieties, and this was possible in some cases. This identification was carried out in pooled cell extracts, and with this information, an in-house database was created with m/z and retention time for each lipid. This in-

house database was used for processing data by MZmine 2 [5]. Glycoceramides were identified based on their accurate mass, MS/MS analysis and retention times. Further verification of majority of the ceramides was possible with authentic standards.

A (semi) quantitation was performed using lipid-class-specific calibration curves (c= 100-5000 ng/mL, with ISTD mixture at c=1250 ng/mL). The calibration standards were as follows: N-oleoyl-D-erythro-sphingosine (Cer d18:1/18:1(9Z))), 1,2-dipalmitoyl-sn-glycero-3-phosphocholine (PC(16:0/16:0)), 1,2-distearoyl-sn-glycero-3-phosphocholine (PC(18:0/18:0)), 1-palmitoyl-2-oleoyl-glycero-3-phosphocholine (PC(16:0/18:1)), 1-stearoyl-2-hydroxy-sn-glycero-3-phosphocholine (LPC(18:0)), 1-oleoyl-2-hydroxy-sn-glycero-3-phosphocholine (LPC(18:1)), cholest-5-en-3b-yl (9Z-octadecenoate) (CE(18:1)), cholest-5-en-3β-yl (9Z,12Z-octadecadienoate) (CE(18:2)), 1-palmitoyl-2-oleoyl-sn-glycero-3-phosphoethanolamine (PE(16:0/18:1)), 1,2,3-trihexadecanoyl-sn-glycerol TG(16:0/16:0/16:0), 1,2,3-trioctadecanoyl-sn-glycerol TG(18:0/18:0/18:0) and N-(9Z-octadecenoyl)-sphing-4-enine-1-phosphocholine (SM(d18:1/18:1(9Z))). The raw variation of the peak areas of internal standards in the samples was, on average, 12.0%, and the RSD of retention times of identified lipids across all samples was on average 0.47%. The RSD of the concentrations of the identified lipids in QC samples and pooled extracts was on average 23%.

Analysis of polar metabolites

To the remaining 75 µL aliquot, 225 µL of ice-cold MeOH (LC-grade, Honeywell) containing the following internal standards (all from Sigma Aldrich): Heptadecanoic acid (5 ppm), DL-valine-d8 (1 ppm), and succinic acid-d4 (1 ppm) was added. The samples were then sonicated in an ice bath for 30 s prior to centrifugation (5500 g, 5 min). 250 µL of the supernatant was transferred to a 2 mL glass autosampler vial. The pellet was stored at −20 °C for protein analysis. The protein content was measured by the Bradford method. The supernatant was dried under a stream of nitrogen at 45 °C. Prior to the mass spectrometry measurements, the samples were derivatized using a two-step procedure. Initially the samples were methoximated by incubating the samples with methoxyamine hydrochloride (25 µL, 20mg/mL in pyridine, Sigma Aldrich) at 45 °C for 1 h. MSTFA (25 µL, Sigma Aldrich) was then added and the samples were incubated for a further 1 h. A retention index standard containing straight chain, even alkanes (n 10-40, 10 µL, Sigma Aldrich) was added. The derivatized samples were analyzed using gas chromatography (Agilent 7890B) coupled to a single quad mass spectrometer (5977B). The metabolites were separated using a 30 m × 0.25 mm (ID) with a film thickness of 0.25 µm HP-5 (Agilent). A guard column (10 m) with an ID of 0.25 mm was used. 1 µL of the sample was injected in splitless mode with an inert glass liner (Agilent) held at a temperature of 240 °C. The GC was set to constant flow mode (1.2 mL/min) using helium (Aga) as the carrier gas. The GC

oven was programed as follows: 50 °C (isothermal for 0.2 min), then 7 °C/min until 240 °C, then 20 °C/min until 300 °C (isothermal for 5 min). The transfer line was held at 260 °C for the whole run. The ion source was set to electron ionization mode and held at 230 °C and the quadrupole at 150 °C. Due to the large number of analytes quantified, the samples were injected twice. The first run quantified the amino acids and the second run quantified all other components. The MSD was set up in select ion monitoring mode to maximize sensitivity. The ions monitored can be found in **(ESM Table 2)**.

Data preprocessing

Lipidomics data processing was performed using open source software MZmine 2.33 [5]. The following steps were applied in the processing: 1) Crop filtering with a m/z range of 350 – 1700 m/z and a RT range of 2.0 to 12 min, 2) Mass detection with a noise level of 1200, 3) Chromatogram builder with a min time span of 0.08 min, min height of 1000 and a m/z tolerance of 0.006 m/z or 10.0 ppm, 4) Chromatogram deconvolution using the local minimum search algorithm with a 70% chromatographic threshold, 0.05 min minimum RT range, 5% minimum relative height, 1200 minimum absolute height, a minimum ratio of peak top/edge of 1 and a peak duration range of 0.08 - 5.0, 5) Isotopic peak grouper with a m/z tolerance of 5.0 ppm, RT tolerance of 0.05 min, maximum charge of 2 and with the most intense isotope set as the representative isotope, 6) Join aligner with a m/z tolerance of 0.008 or 10.0 ppm and a weight for of 2, a RT tolerance of 0.1 min and a weight of 1 and with no requirement of charge state or ID and no comparison of isotope pattern, 7) Peak list row filter with a minimum of 12 peaks in a row (= 10% of the samples), 8) Gap filling using the same RT and m/z range gap filler algorithm with an m/z tolerance of 0.006 m/z or 10.0 ppm, 9) Identification of lipids using a custom database search with an m/z tolerance of 0.006 m/z or 10.0 ppm and a RT tolerance of 0.1 min, 10) Normalization using lipid-class-specific internal standards and (semi) quantitation with lipid-class-specific calibration curves, 11) Normalization with total protein amount 12) Data imputation of missing values were done with half of the row's minimum.

The GC-QMS data was processed in MassHunter Quant (v8, Agilent technologies) The peaks were manually checked and corrected if needed for correct integration. Quantification was performed using the ion listed **(ESM Table 2)**. Standard curves were used to quantify each metabolite using the assigned internal standards. Metabolites which had a CV greater than 30% in the pooled QC sample or fell below the limit of quantification were excluded from subsequent analysis.

Statistical methods
Homogeneity of the samples were assessed by principal component analysis (PCA) which was performed using *'prcomp'* function included in the *'stats'* package of R. The scores of the observation falling outside the 95% confidence interval were considered as outlier.

The effect of different factors such as age, gender, conditions and their interactions on the lipidomics dataset was evaluated. The data was centered to zero mean and unit variance. The relative contribution of each factor to the total variance in the dataset was estimated by fitting a linear regression model, where the normalized intensities of metabolites were regressed to the factor of interest, and thereby median marginal coefficients (R^2) were estimated **(ESM Fig. 4)**. This analysis was performed using *'scater'* package.

Sparse Partial least squares Discriminant Analysis (sPLS-DA) [6] models comparing P1Ab *vs.* CTRL, PT1D *vs.* CTRL, and PT1D *vs.* P1Ab groups, paired at 12, 24 and 36 months were developed and Variable Importance in Projection (VIP) scores [7] of the features/metabolites were estimated. sPLS-DA modeling was performed using the '*splsda*' function coded in the *'mixOmics v6.3.2'* package. sPLS-DA models were cross-validated [8] by 7-fold cross-validation and models diagnostics were generated using *'perf'* function. The multivariate analysis was followed by univariate; Two-Sample t-testing using the *'t.test'* function was applied to compare the mean differences in the metabolite intensities between P1Ab *vs.* CTRL, PT1D *vs.* CTRL, and PT1D *vs.* P1Ab groups. Together, multi- and univariate analysis was used for selection of metabolites altered between these subgroups at a particular age. All metabolites that passed one or more criteria for variable selection, *i.e.*, with sPLS-DA model area under the ROC curve (AUC) >= 0.65; RC (>± 0.05), VIP scores > 1 and/or (T-test; p-value < 0.05) were listed as altered. Multiple testing was performed and the nominal p-values (T-test) were subjected to FDR correction. The features/metabolites did not pass the threshold (FDR corrected p-values < 0.05) which might be due to small sample size of these subgroups, analyzed at a particular time-point.

Spearman correlation was applied to identify association between plasma and cellular metabolite levels in CTRL, P1Ab and PT1D groups. Spearman's correlation coefficient (ρ) was calculated using the *'rcorr'* function implemented in the *'Hmisc'* package. P-values were subjected to False Discovery Rates (FDR) adjustment using '*p-adjust*'. Loess regression was used for

the interpolation of the metabolite intensities along 12, 24 and 36 months of age. It was performed using *'loess'* function deployed in the 'stats' package.

'Heatmap.2', *'boxplot'*, *'beanplot'*, *'gplot'*, and *'ggplot2'* libraries/packages were used for data visualization.

Pathway overrepresentation analysis (POA) was performed using the MetaboAnalyst 4.0 web platform [9] using the 'Pathway Analysis' module. Those metabolites altered between different subgroups were listed and mapped to the human metabolic network as a background; 'relative-betweenness Centrality' was selected for 'pathway topology analysis', and global hypergeometric test (GHT) was performed. GHT estimated the relative significance of the overrepresented pathways against the background KEGG pathways [10] for *Homo sapiens*. The metabolic subsystems/pathways with (FDR < 0.05) is reported in (**Fig. 4**). The Pathway Impact Scores (PIS) were estimated by the metabolomics pathway analysis (MetPA) tool [11] encoded in MetaboAnalyst 4.0 [9].

Meta-analysis of transcriptomics datasets and genome-scale metabolic modeling

Genome-scale metabolic modelling (GSMM) is a constraint-based mathematical modelling approach that integrates biochemical, genetic and genomic informations within a computational framework [12-15]. It is used to study metabolic genotype-phenotype relationship of an organism. They are efficient tools for prediction of growth in living cells/tissues exposed to different nutrients [16, 17]. The structure of genome-scale model (GEM) provides scaffolds for integration of different types of omics data such as transcriptome, proteome and metabolome/fluxome [18]. Several algorithms were designed that allow integration and contextualization of GEMs (i.e. to constrained the metabolic reaction bounds with the experimental data or conditions), based on expression datasets [19].

The feasibility of a particular metabolic reaction(s) to be included or discarded in the draft GEM model was evaluated. The polar metabolites and the lipid intensities from this study were used to estimate the confidence score of a metabolite in a reaction [20], that is either included or discarded in the draft model. By applying this strategy, condition-specific PBMC models for PT1D, P1Ab and CTRL were developed. Quality control (QC) or sanity checks were performed on the draft models [21-23]. In addition, the models were tested for their ability to carry out basic metabolic tasks [24, 25]. The blocked reactions were removed before simulations.

Reporter metabolites [26, 27] were predicted by using '*reporterMetabolites*' algorithm/function coded in the RAVEN 2.0 suite [23]. Mixed integer linear programming (MILP) and linear programming (LP) was performed using *'MOSEK 8'* solver (licensed for the academic user) integrated in the RAVEN 2.0 toolbox [23]. The lower and/or upper bound of an exchange reaction or the uptake rates of a PBMC model were derived from the metabolite concentrations using *'conc2Rate'* function from COnstraint-Based Reconstruction and Analysis Toolbox (Cobra toolbox v3.0) [22]. Flux Enrichment Analysis (FEA) was performed using *'FEA'* of Cobra toolbox v3.0. All the simulations were performed in MATLAB 2017b (Mathworks, Inc., Natick, MA, USA).

**Lipids**	**P1Ab** (p-values)	**PT1D** (p-values)
Cer(d18:1/24:0)	0.286	0.03
PC(36:3)	0.036	0.309
TG(45:0)	0.025	0.135
TG(47:0)	0.017	0.379
TG(48:0)	0.041	0.013
TG(49:1)	0.029	0.614
TG(50:0)	0.032	0.053
TG(50:1)	0.016	0.007
TG(16:0/16:0/16:0)	0.302	0.011
TG(18:0/18:1/20:4)	0.7	0.03
TG(18:1/12:0/18:1) or TG(18:2/16:0/14:0)	0.119	0.022
TG(18:1/18:1/16:0)	0.791	0.02
TG(18:2/18:1/18:1)	0.882	0.02
TG(48:1)	0.194	0.033
TG(48:3)	0.9	0.042
TG(49:2)	0.209	0.045
TG(50:2)	0.066	0.012
TG(52:2)	0.305	0.018
TG(52:3)	0.414	0.005
TG(53:2)	0.226	0.016
TG(54:6)	0.932	0.007

ESM Table 1. Lipids altered before and after seroconversion (pre- vs. post-seroconversion analysis).

Metabolite name	Derivitzed metabolite	Retention time	Retention index	Quantification Ion	Qualifier Ion 1	Qualifier Ion 2
Alanine	Alanine 3TMS	17.116	1431	174.1	248.1	304.1
Aspartic acid	Aspartic 3TMS	18.758	1517	232.1	176.1	293.1
Citric acid	Citric acid 4TMS	23.649	1825	273.1	363.1	465.1
Cystine	Cystine 4TMS	30.075	2103	218.1	146.0	411.1
Dihydroxyacetoncephosphate	Dihydroxyacetoncephosphate	20.441	1604	315.1	299.1	400.1
Fructose-6-phosphate	Fructose-6-phosphate	30.075	2294	315.1	217.1	459.2
Gluctose-6-phosphate	Gluctose-6-phosphate	30.189	2302	387.2	357.1	471.2
Glutamic acid	Glutamic acid 3TMS	20.469	1605	246.1	230.1	348.1
Lysine	Lysine 4TMS	25.107	1931	174.1	317.2	434.2
Myristic acid	Myristic acid TMS	24.064	1855	285.3	129.1	300.2
Ornithine	Ornithine 3TMS	22.576	1746	174.1	348.2	186.1
Oxoproline	Oxoproline 2TMS	18.747	1516	156.1	230.1	258.1
Palmitic acid	Palmitic acid TMS	26.859	2059	313.2	132.0	328.3
Pentanedioic acid	Pentanedioic acid(imino) 2TMS	19.724	1567	147.1	198.0	304.1
Phenylalanine	Phenylalanine 3TMS	20.53	1608	218.1	192.1	266.1
Phosphoenolpyruvate	Phosphoenolpyruvate	20.121	1587	369.1	299.1	384.0
Proline	Proline 2TMS	14.637	1303	142.1	216.1	244.1
Serine	Serine 3TMS	15.845	1366	204.1	218.1	278.1
Stearic acid	Stearic acid TMS	30.189	2302	341.3	117.0	356.3
Threonine	Threonine 3TMS	16.339	1391	218.1	117.1	291.1
Tyrosine	Tyrosine 3TMS	25.331	1947	218.1	280.1	382.2
Unknown BCAA 1	BCAA 1	11.897	1161	86.1	75.0	188.0
Unknown BCAA 2	BCAA 2	12.314	1183	86.1	75.0	188.0
Unknown BCAA 3	BCAA 3	12.734	1204	86.1	75.0	188.0
Valine	Valine 2TMS	10.651	1096	144.1	100.1	218.1

ESM Table 2. List of polar metabolites analyzed.

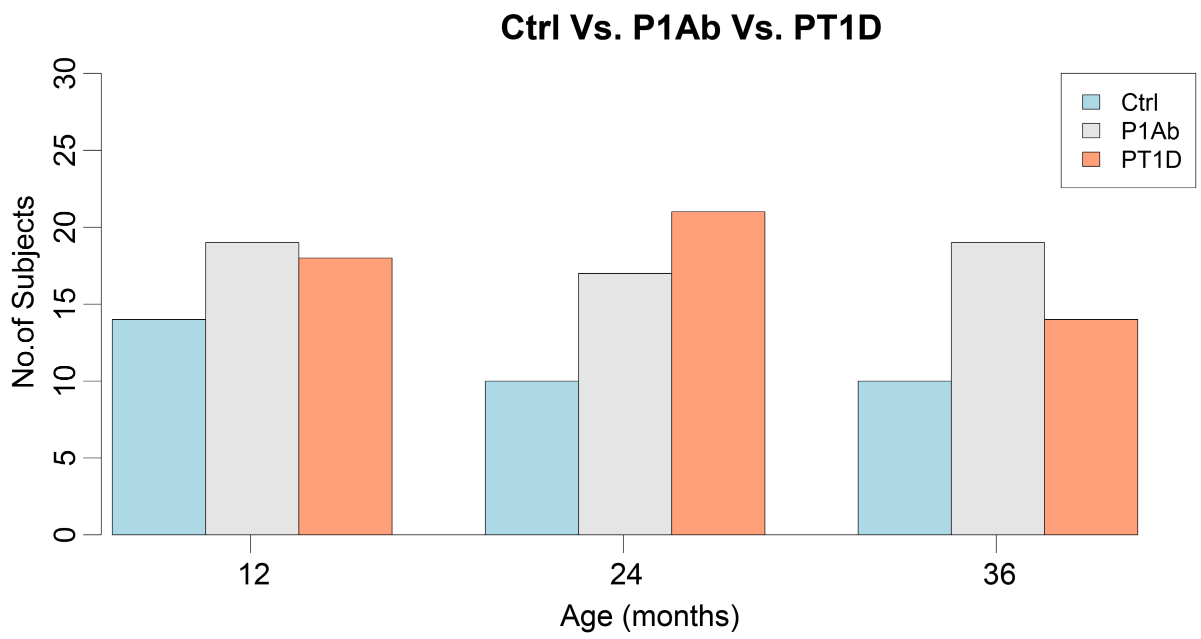

ESM Fig.1 Bar plot showing the number of subjects included in the, (i) *Ctrl (CTRL)* group: children who remained autoantibody negative (Ab^{-}) during the follow-up. (ii) *P1Ab* group: children who developed at least one islet autoantibody (Ab^{+}) but were not diagnosed with type 1 diabetes (T1D), during the follow-up. (iii) *PT1D* group: comprises of children seroconverted to multiple islet autoantibodies (Ab^{++}) and later developed T1D. The subjects are ordered by their age groups.

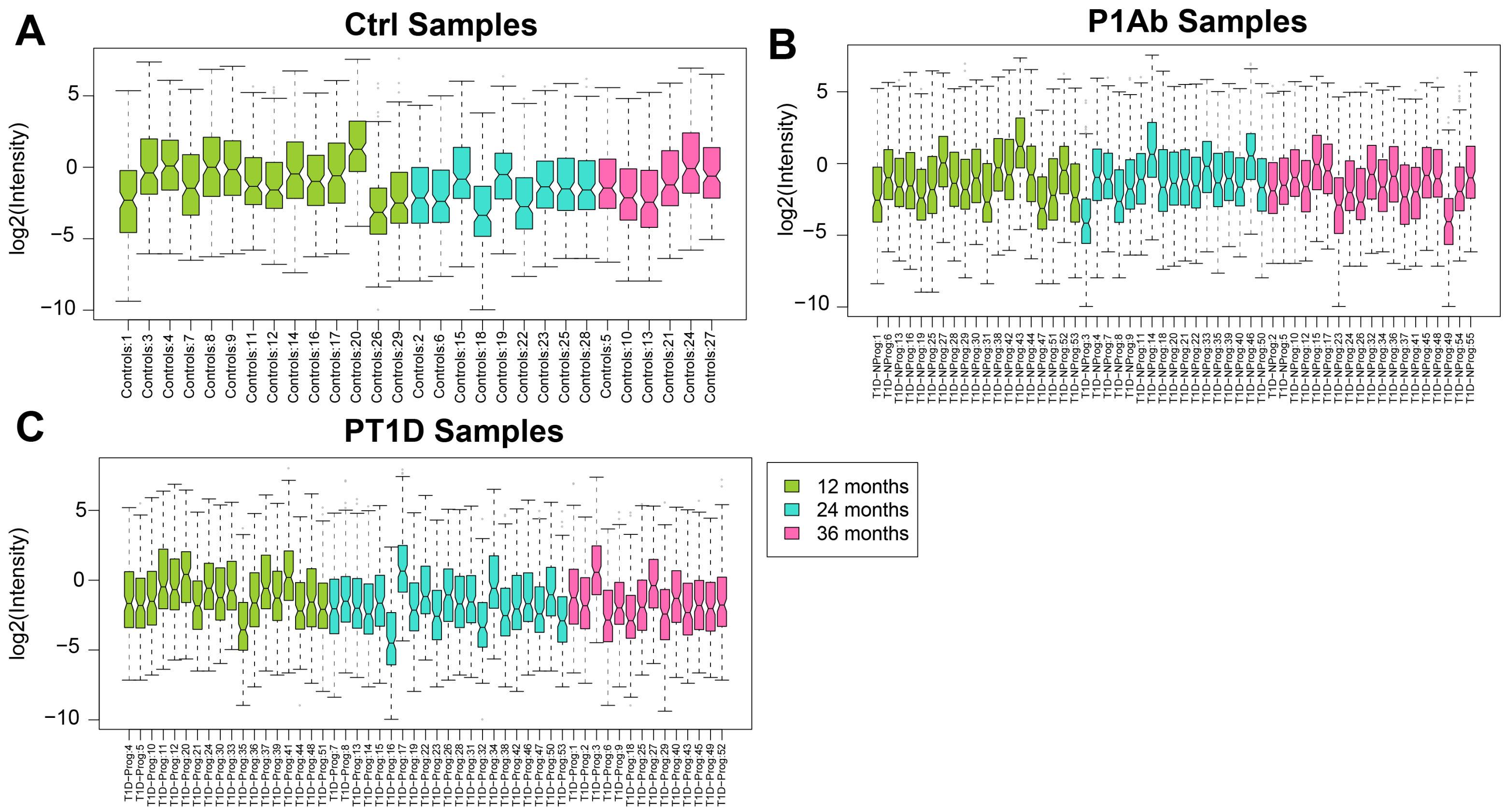

ESM Fig.2 (A-C) Log normalized intensities of the lipids measured in *Ctrl, P1Ab* and *PT1D* groups. The subjects are colored and ordered by their age groups. The outliers are marked by grey dots.

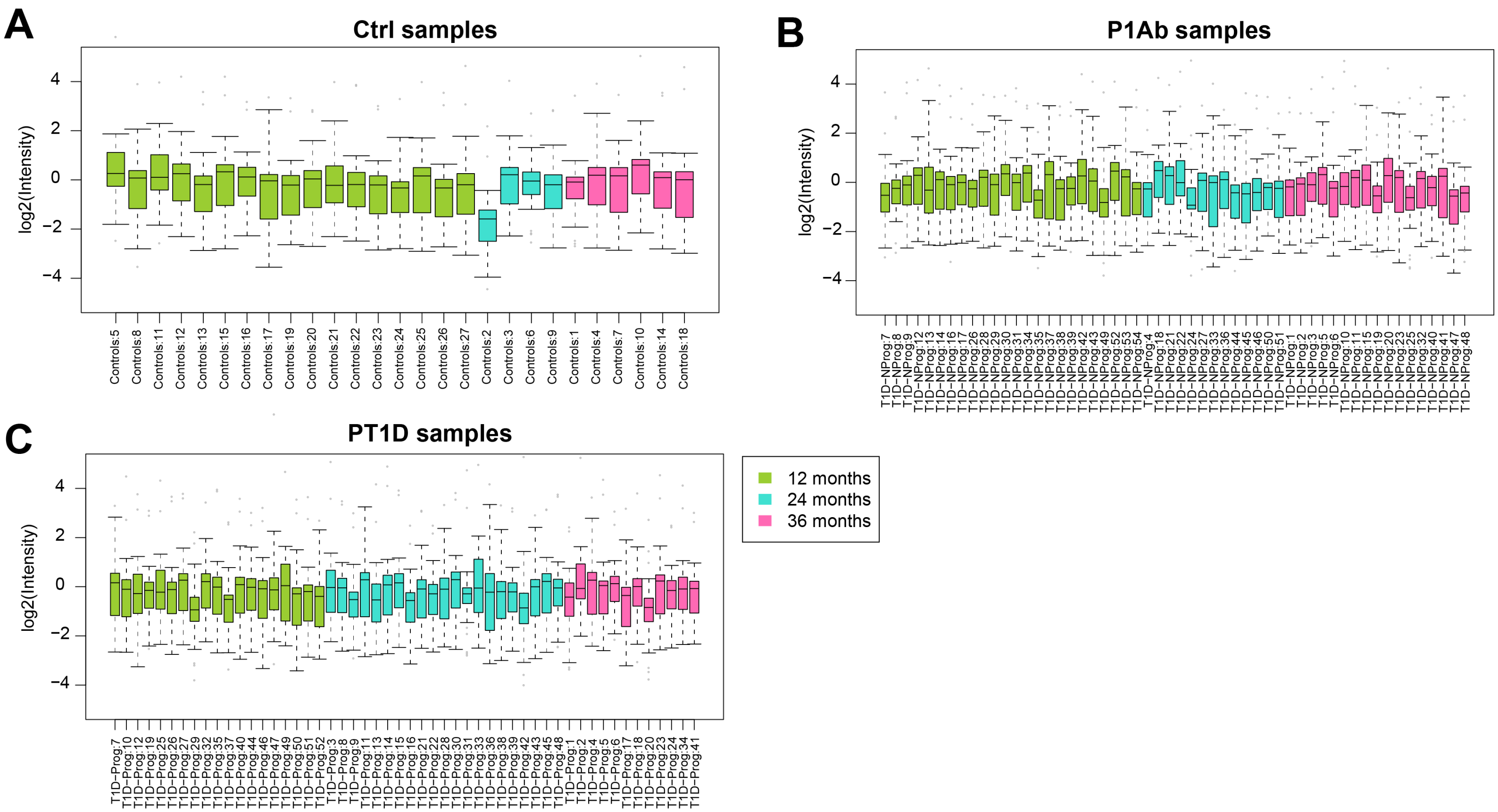

ESM Fig.3 (A-C) Log normalized intensities of the polar metabolites measured in *Ctrl, P1Ab* and *PT1D* groups. The subjects are colored and ordered by their age groups. The outliers are marked by grey dots.

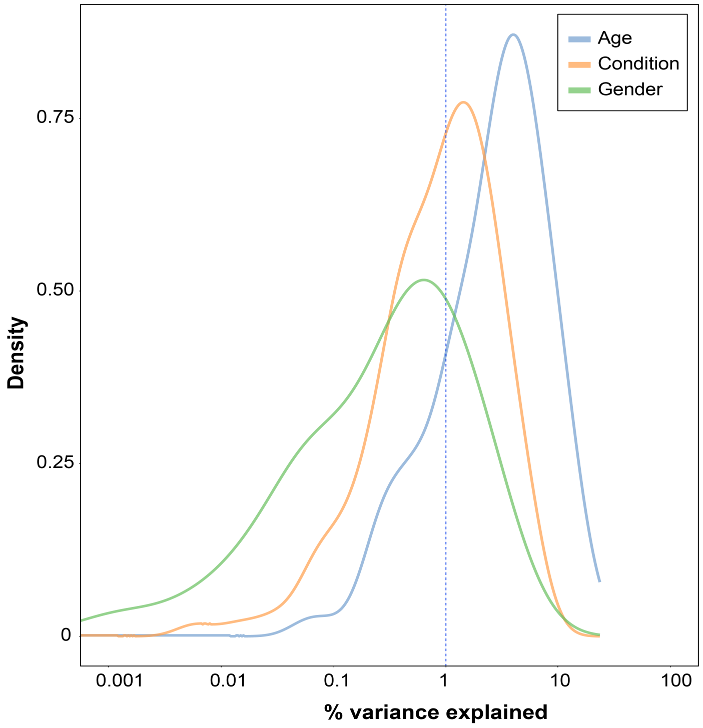

ESM Fig.4 Factors and sources of variation in the metabolomics datasets, displaying a density plot of the metabolite-wise marginal R^2 values for the factors. Effects of different factors such as age, gender and study group status on lipidomics data were evaluated. The data were centered to zero mean and unit variance. The relative contribution of each factor (experimental variable) to the total variance in the dataset was estimated by fitting the linear model regression model, where the normalized intensities of metabolites regressed to the factor of interest, and thereby estimating the median marginal coefficients (R^2). This analysis was performed using package 'scater'.

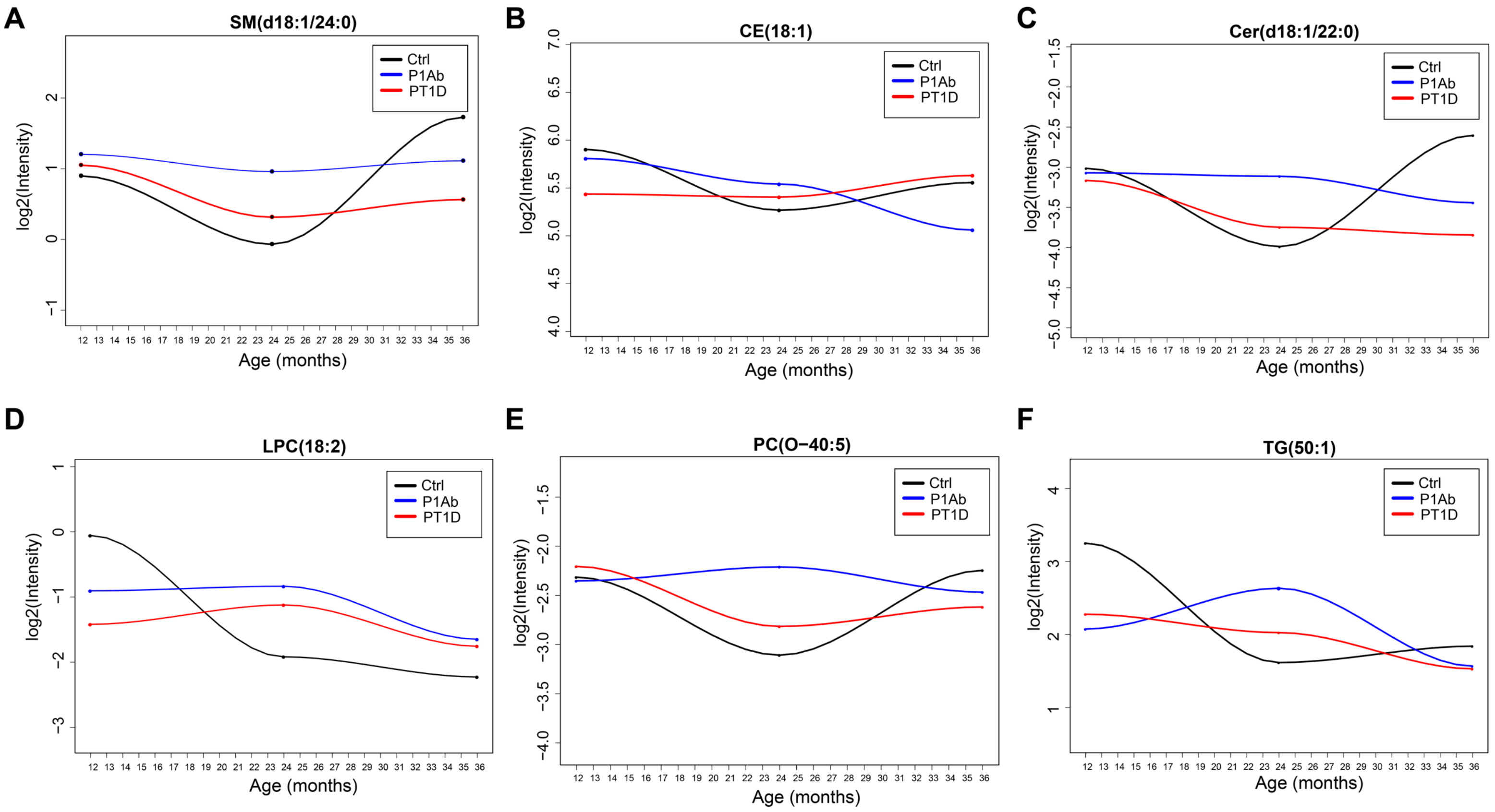

ESM Fig.5 Selected lipid profiles that were altered in *Ctrl*, *P1Ab* and *PT1D* groups, during the 36-months follow-up. (A-F) The log mean intensities of sphingomyelin (SMs), cholesterol esters (CEs), ceramides (Cer), lysophosphatidylcholine (LPCs), phosphatidylcholine (PCs), and triacylglycerides (TGs) are shown along the age (months). Loess regression was used for the interpolation of the data points.

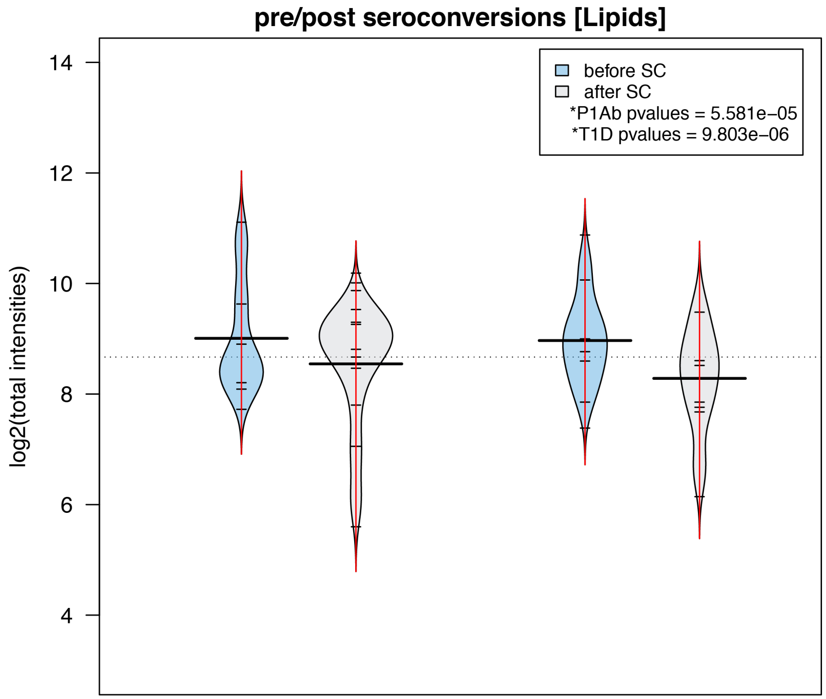

ESM Fig.6 Log intensities of the total lipids before (light blue) and after (light gray) the seroconversion (SC), in *P1Ab* and *PT1D* groups. Down-regulation in the intensities of the total lipids in the PBMCs were observed after seroconversion (SC), in *P1Ab* (median age of SC, 24 months) (p=5.581e-05) and *PT1D* (median age of SC, 14 months) (p=9.803-06).

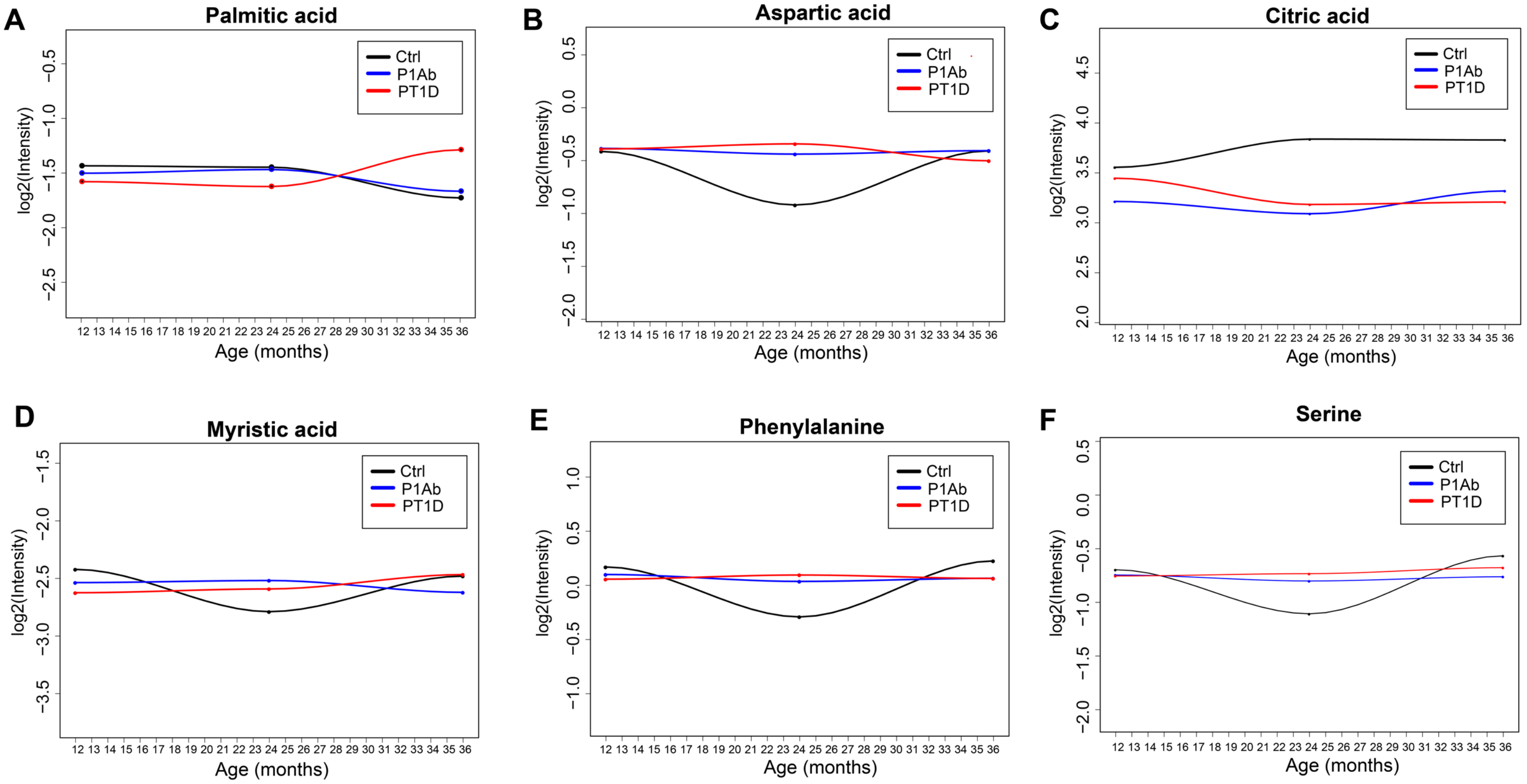

ESM Fig.7 Selected profiles of polar metabolites that were altered in *Ctrl*, *P1Ab*, and *PT1D* groups, during the 36-months follow-up. (A-F) The log mean intensities of palmitic acid, aspartic acid, citric acid, myristic acid, phenylalanine, and serine are shown along the age (months). Loess regression was used for the interpolation of the data points.

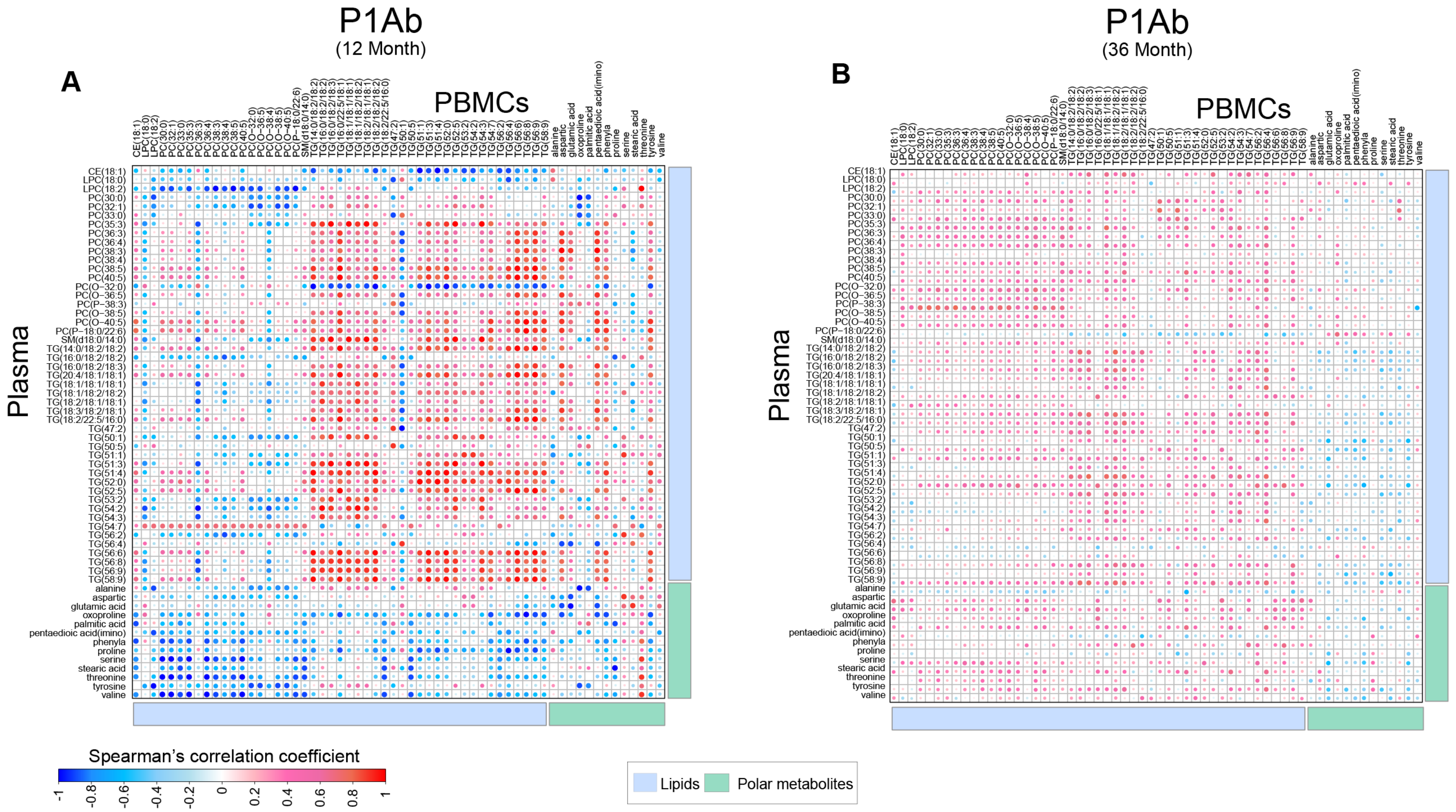

ESM Fig.8 Heatmap showing Spearman's correlation between plasma and cellular (PBMCs) metabolite intensities in *P1Ab* at 12 and 36-months of follow-up. Red, blue and white color suggests positive, inverse and no correlation respectively.

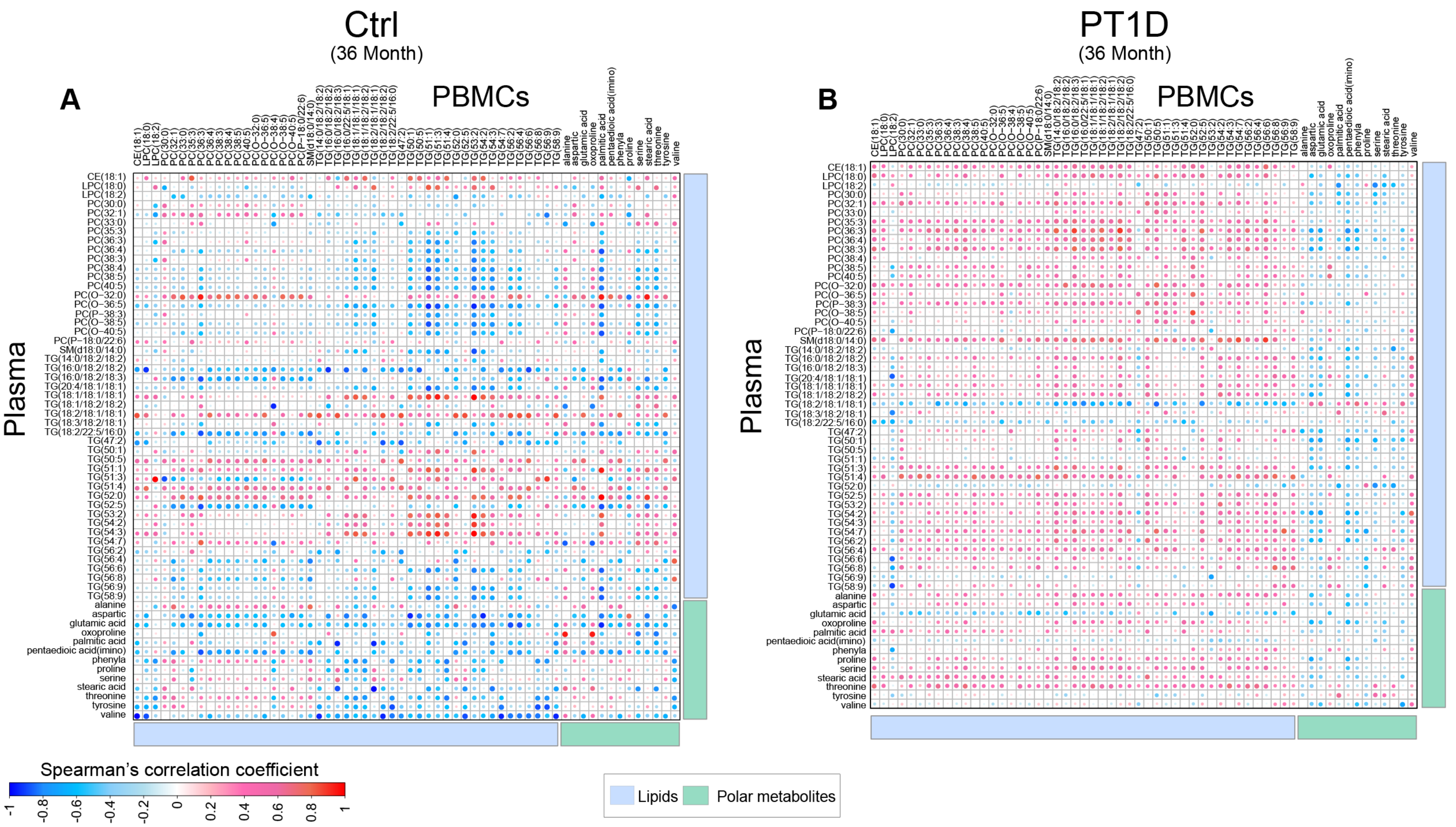

ESM Fig.9 Heatmap showing Spearman's correlation between plasma and cellular (PBMCs) metabolite intensities in *Ctrl* and *PT1D* at 36-months of follow-up. Red, blue and white color suggests positive, inverse and no correlation respectively.

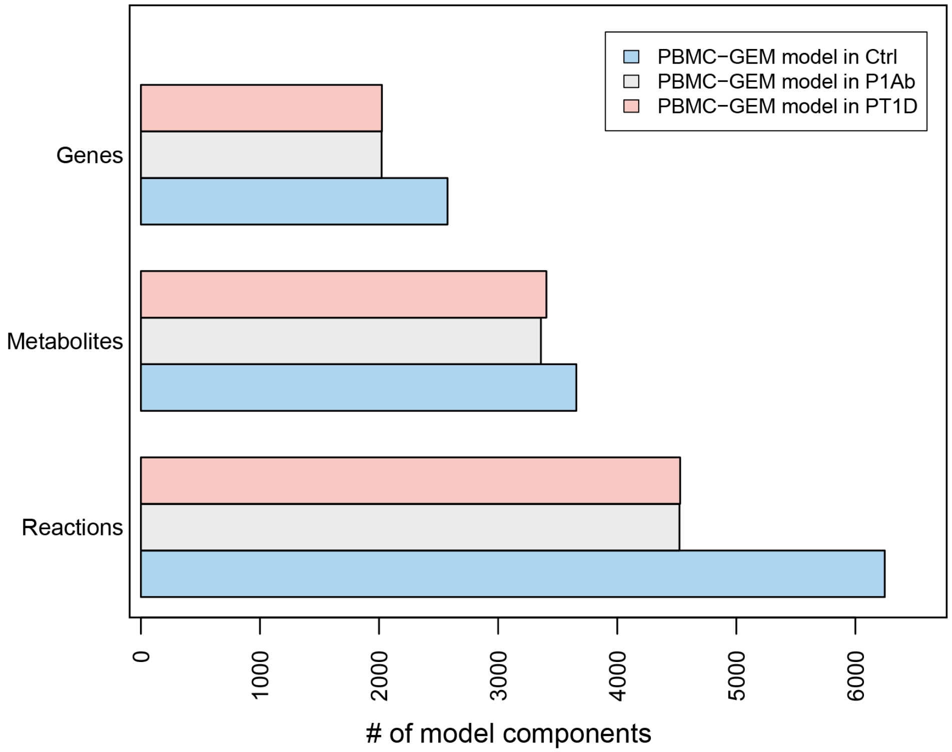

Figure.S10

ESM Fig.10 Genome-scale metabolic models for PBMCs. The total numbers of genes, metabolites and reactions included in the PBMC models for *Ctrl*, *P1Ab* and *PT1D*, developed in the study is shown.

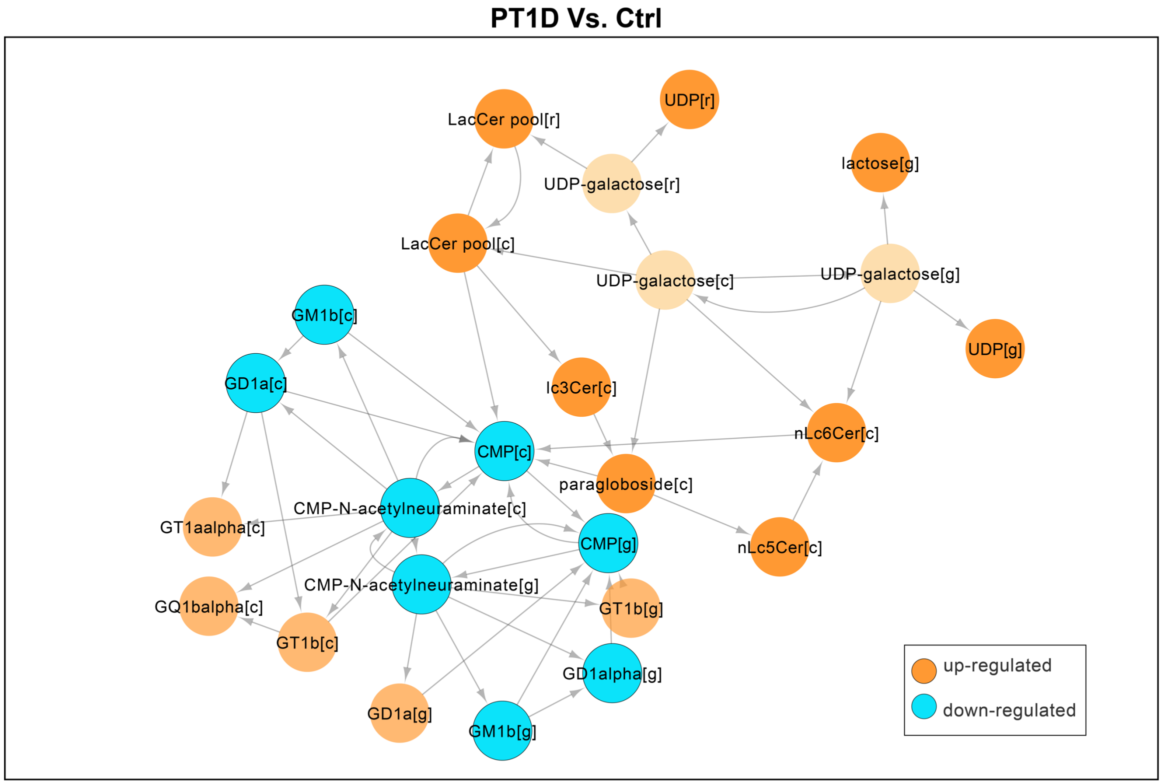

ESM Fig.11 Reporter metabolites of PBMCs, based on genome-scale metabolic modeling, that were significantly changed (FDR < 0.05) in *PT1D vs. Ctrl.*

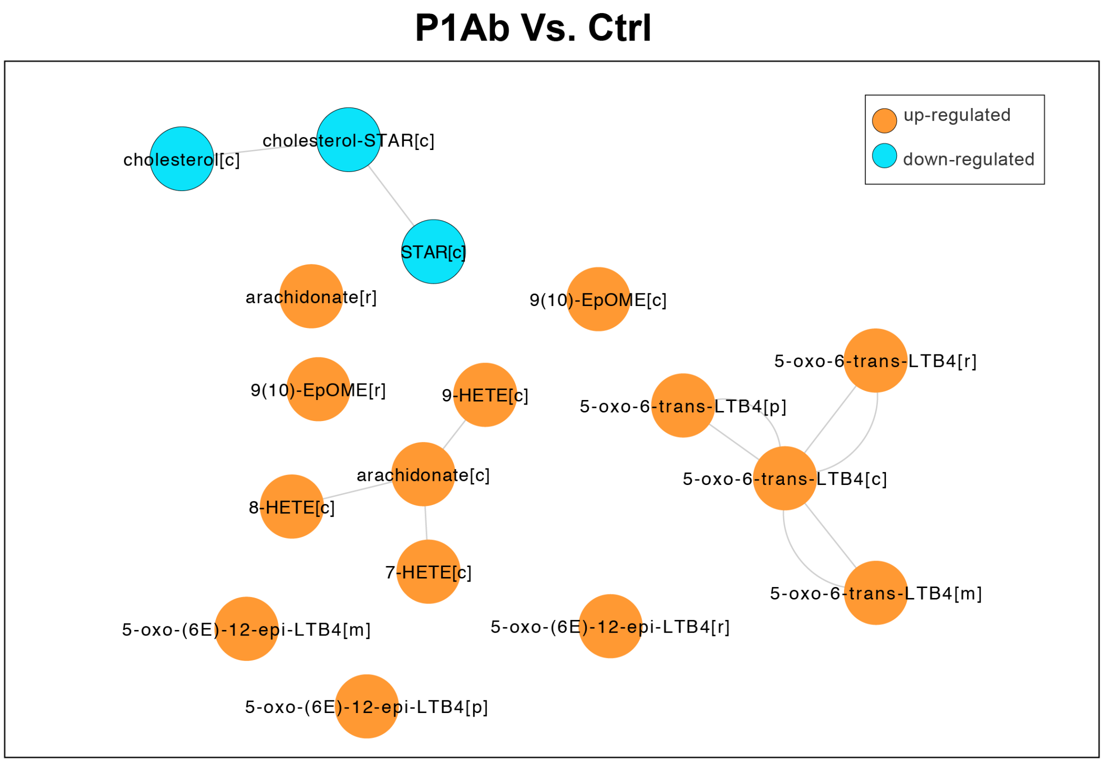

ESM Fig.12 The plot shows reporter metabolites of PBMCs that were significantly changed (FDR < 0.05) in *P1Ab vs. Ctrl*.

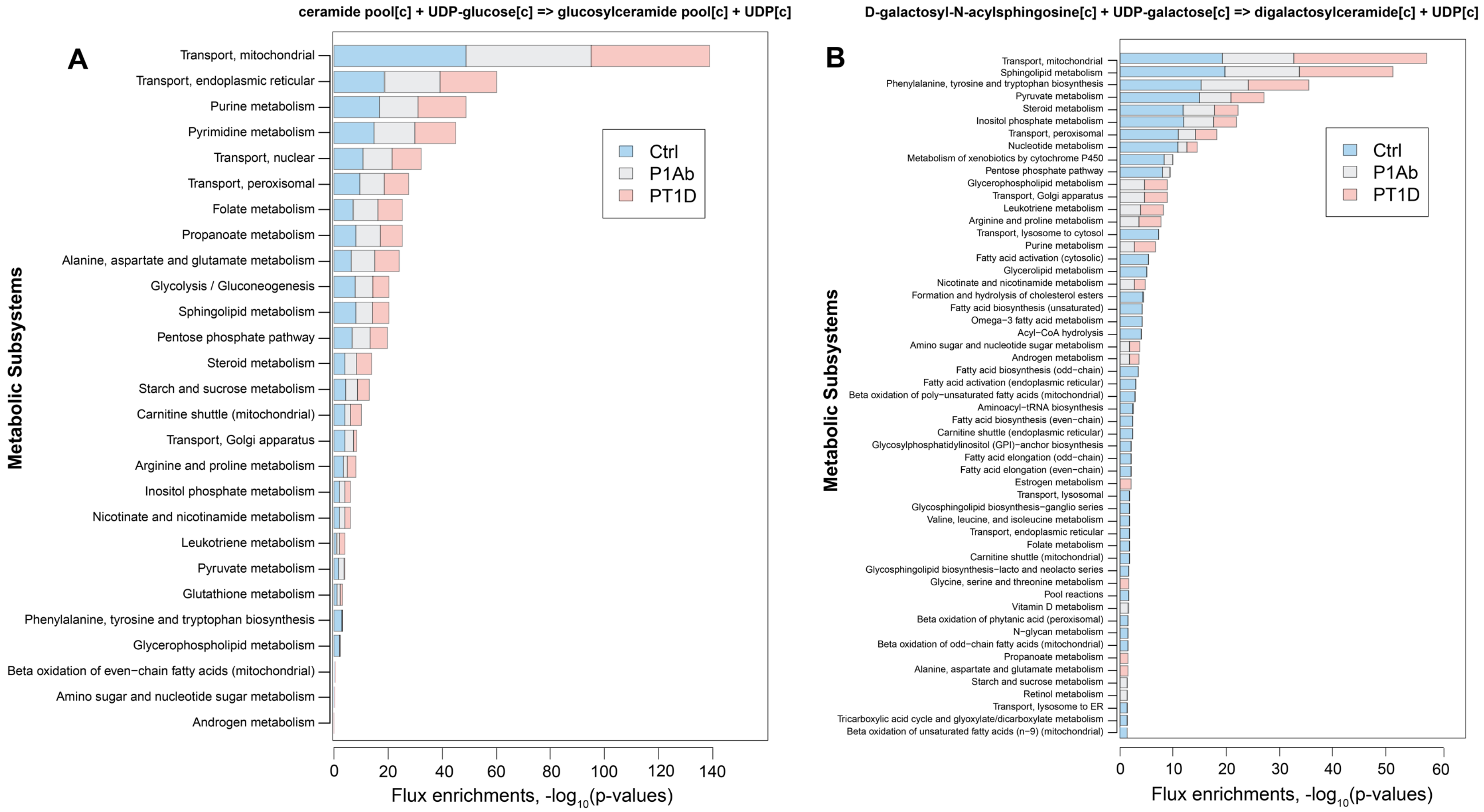

ESM Fig.13 Flux enrichments in the metabolic subsystems. Stacked bar plots showing cumulative (-log₁₀) q-values of the enriched subsystems. Enrichments of metabolic subsystems in PBMCs models for T1D progressors and nonprogressors when, (A) production of glucosylceramide is maximized, (B) production of digalactosylceramide from D-galactosyl-N-acylsphingosine is maximized.

References

[1] Bradford MM (1976) A rapid and sensitive method for the quantitation of microgram quantities of protein utilizing the principle of protein-dye binding. Anal Biochem 72: 248-254

[2] Folch J, Lees M, Sloane Stanley G (1957) A simple method for the isolation and purification of total lipids from animal tissues. J biol Chem 226(1): 497-509

[3] Nygren H, Seppanen-Laakso T, Castillo S, Hyotylainen T, Oresic M (2011) Liquid chromatography-mass spectrometry (LC-MS)-based lipidomics for studies of body fluids and tissues. Methods Mol Biol 708: 247-257. 10.1007/978-1-61737-985-7_15

[4] Pedersen HK, Forslund SK, Gudmundsdottir V, et al. (2018) A computational framework to integrate high-throughput '-omics' datasets for the identification of potential mechanistic links. Nat Protoc 13(12): 2781-2800. 10.1038/s41596-018-0064-z

[5] Pluskal T, Castillo S, Villar-Briones A, Oresic M (2010) MZmine 2: modular framework for processing, visualizing, and analyzing mass spectrometry-based molecular profile data. BMC Bioinform 11: 395. 10.1186/1471-2105-11-395

[6] Le Cao KA, Boitard S, Besse P (2011) Sparse PLS discriminant analysis: biologically relevant feature selection and graphical displays for multiclass problems. BMC Bioinformatics 12: 253. 10.1186/1471-2105-12-253

[7] Farrés M, Platikanov S, Tsakovski S, Tauler R (2015) Comparison of the variable importance in projection (VIP) and of the selectivity ratio (SR) methods for variable selection and interpretation. J Chemom 29(10): 528-536

[8] Westerhuis JA, Hoefsloot HC, Smit S, et al. (2008) Assessment of PLSDA cross validation. Metabolomics 4(1): 81-89

[9] Chong J, Soufan O, Li C, et al. (2018) MetaboAnalyst 4.0: towards more transparent and integrative metabolomics analysis. Nucleic Acids Res 46(W1): W486-W494. 10.1093/nar/gky310

[10] Kanehisa M, Goto S, Sato Y, Kawashima M, Furumichi M, Tanabe M (2013) Data, information, knowledge and principle: back to metabolism in KEGG. Nucleic acids research 42(D1): D199-D205

[11] Xia J, Wishart DS (2010) MetPA: a web-based metabolomics tool for pathway analysis and visualization. Bioinformatics 26(18): 2342-2344. 10.1093/bioinformatics/btq418

[12] Orth JD, Thiele I, Palsson BØ (2010) What is flux balance analysis? Nature biotechnology 28(3): 245-248

[13] Price ND, Reed JL, Palsson BØ (2004) Genome-scale models of microbial cells: evaluating the consequences of constraints. Nature Reviews Microbiology 2(11): 886-897

[14] O'Brien EJ, Monk JM, Palsson BO (2015) Using genome-scale models to predict biological capabilities. Cell 161(5): 971-987

[15] Bordbar A, Monk JM, King ZA, Palsson BO (2014) Constraint-based models predict metabolic and associated cellular functions. Nature Reviews Genetics 15(2): 107-120

[16] O'Brien EJ, Lerman JA, Chang RL, Hyduke DR, Palsson BO (2013) Genome-scale models of metabolism and gene expression extend and refine growth phenotype prediction. Mol Syst Biol 9: 693. 10.1038/msb.2013.52

[17] Förster J, Famili I, Fu P, Palsson BØ, Nielsen J (2003) Genome-scale reconstruction of the Saccharomyces cerevisiae metabolic network. Genome research 13(2): 244-253

[18] Blazier AS, Papin JA (2012) Integration of expression data in genome-scale metabolic network reconstructions. Frontiers in physiology 3: 299
[19] Becker SA, Palsson BO (2008) Context-specific metabolic networks are consistent with experiments. PLoS Comput Biol 4(5): e1000082
[20] Agren R, Bordel S, Mardinoglu A, Pornputtapong N, Nookaew I, Nielsen J (2012) Reconstruction of genome-scale active metabolic networks for 69 human cell types and 16 cancer types using INIT. PLoS Comput Biol 8(5): e1002518
[21] Thiele I, Palsson BO (2010) A protocol for generating a high-quality genome-scale metabolic reconstruction. Nat Protoc 5(1): 93-121. 10.1038/nprot.2009.203
[22] Heirendt L, Arreckx S, Pfau T, et al. (2017) Creation and analysis of biochemical constraint-based models: the COBRA Toolbox v3. 0. arXiv preprint arXiv:171004038
[23] Wang H, Marcisauskas S, Sanchez BJ, et al. (2018) RAVEN 2.0: A versatile toolbox for metabolic network reconstruction and a case study on Streptomyces coelicolor. PLoS Comput Biol 14(10): e1006541. 10.1371/journal.pcbi.1006541
[24] Mardinoglu A, Agren R, Kampf C, et al. (2013) Integration of clinical data with a genome-scale metabolic model of the human adipocyte. Mol Syst Biol 9: 649. 10.1038/msb.2013.5
[25] Mardinoglu A, Agren R, Kampf C, Asplund A, Uhlen M, Nielsen J (2014) Genome-scale metabolic modelling of hepatocytes reveals serine deficiency in patients with non-alcoholic fatty liver disease. Nature communications 5
[26] Cakir T, Patil KR, Onsan Z, Ulgen KO, Kirdar B, Nielsen J (2006) Integration of metabolome data with metabolic networks reveals reporter reactions. Mol Syst Biol 2: 50. 10.1038/msb4100085
[27] Patil KR, Nielsen J (2005) Uncovering transcriptional regulation of metabolism by using metabolic network topology. Proceedings of the National Academy of Sciences of the United States of America 102(8): 2685-2689. 10.1073/pnas.0406811102
